# Development of molecular clamp stabilized hemagglutinin vaccines for Influenza A viruses

**DOI:** 10.1038/s41541-021-00395-4

**Published:** 2021-11-08

**Authors:** Christopher L. D. McMillan, Stacey T. M. Cheung, Naphak Modhiran, James Barnes, Alberto A. Amarilla, Helle Bielefeldt-Ohmann, Leo Yi Yang Lee, Kate Guilfoyle, Geert van Amerongen, Koert Stittelaar, Virginie Jakob, Celia Lebas, Patrick Reading, Kirsty R. Short, Paul R. Young, Daniel Watterson, Keith J. Chappell

**Affiliations:** 1 School of Chemistry and Molecular Biosciences, The University of Queensland, St Lucia, QLD 4072 Australia; 2 WHO Collaborating Centre for Reference and Research on Influenza, Peter Doherty Institute for Infection and Immunity, Melbourne, VIC 3000 Australia; 3 Australian Infectious Diseases Research Centre, Global Virus Network Centre of Excellence, Brisbane, QLD 4072 and 4029 Australia; 4 School of Veterinary Science, The University of Queensland Gatton Campus, Gatton, QLD 4343 Australia; 5 Viroclinics Xplore, Landerd Campus, Nistelrooise Baan 3, 5374 RE Schaijk, The Netherlands; 6 Vaccine Formulation Institute, Chemin des Aulx 14, 1228 Plan-Les-Ouates, Geneva, Switzerland; 7 Department of Microbiology and Immunology, Peter Doherty Institute for Infection and Immunity, The University of Melbourne, Parkville, VIC 3000 Australia; 8 The Australian Institute of Biotechnology and Nanotechnology, The University of Queensland, St Lucia, QLD 4072 Australia

**Keywords:** Protein vaccines, Influenza virus

## Abstract

Influenza viruses cause a significant number of infections and deaths annually. In addition to seasonal infections, the risk of an influenza virus pandemic emerging is extremely high owing to the large reservoir of diverse influenza viruses found in animals and the co-circulation of many influenza subtypes which can reassort into novel strains. Development of a universal influenza vaccine has proven extremely challenging. In the absence of such a vaccine, rapid response technologies provide the best potential to counter a novel influenza outbreak. Here, we demonstrate that a modular trimerization domain known as the molecular clamp allows the efficient production and purification of conformationally stabilised prefusion hemagglutinin (HA) from a diverse range of influenza A subtypes. These clamp-stabilised HA proteins provided robust protection from homologous virus challenge in mouse and ferret models and some cross protection against heterologous virus challenge. This work provides a proof-of-concept for clamp-stabilised HA vaccines as a tool for rapid response vaccine development against future influenza A virus pandemics.

## Introduction

Influenza viruses are medically significant members of the *Orthomyxoviridae* family, causing an estimated 3–5 million severe infections resulting in 290,000–650,000 deaths annually^1^. In addition to regular seasonal outbreaks caused by strains circulating within the human population, there have been numerous influenza virus pandemics in the past century resulting from a new influenza strain entering the human population, with the most recent occurring in 2009^2–6^. Of additional concern are the avian influenza (HPAI) viruses such as H5N1, H7N9, and H9N2 that have caused sporadic spillover events with high fatality rates (~30–50%)^7–9^. While these viruses do not currently exhibit efficient human-to-human transmission, the potential for a novel virus that does possess such transmissibility is significant, which could result in another global pandemic^8–13^. A large reservoir of novel avian influenza viruses is present in aquatic birds, further highlighting the pandemic potential of influenza viruses^14,15^.

Current influenza vaccine production mostly involves virus propagation in embryonated chicken eggs, before chemical inactivation and “splitting” of the viral components using surfactants. The vaccines are delivered as multivalent formulations containing antigen from three to four circulating strains including two influenza A and one or two circulating influenza B viruses^16^. However, these vaccines have shown suboptimal efficacy, ranging from just 10–60% over the last fifteen years^17^. As such, these must be updated and readministered annually. However, due to the lead time required to formulate a new egg-based vaccine, potential difficulties generating a virus that replicates effectively in embryonated chicken eggs and limitations in availability of eggs, these approaches are not well suited to a pandemic response^18^. To counteract the threat of a potential influenza pandemic, either a universal influenza vaccine or technology to be able to rapidly respond to the novel virus and develop a vaccine is required.

The antibodies induced by current vaccines are primarily directed towards the hemagglutinin (HA) protein, which is the virus glycoprotein responsible for binding to host cell receptors and initiating membrane fusion^19–25^. HA consists of two domains: the head and the stem. The head domain contains a large degree of sequence diversity (34–59% between subtypes) and rapidly accumulates mutations, which is a major factor contributing to the lack of vaccine efficacy and need for annual updating^26^. Conversely, the stem domain of HA is structurally and functionally constrained and therefore more conserved across subtypes. A number of monoclonal antibodies against the HA stem, which primarily afford protection via Fc-mediated effector functions rather than virus neutralisation, have been shown to be broadly reactive and protective against highly divergent influenza viruses^27–31^. As a result, a good proportion of the next generation of universal influenza virus vaccine candidates are aimed at stimulating broadly reactive stem-specific antibodies. Most approaches have used recombinant protein technology, utilising trimerisation domains such as the bacteriophage T4 fibritin foldon or the leucine zipper GCN4 to constrain HA in its pre-fusion conformation and retain key stem domain epitopes that are not available for binding in the post-fusion conformation. While these approaches have increased stem-specific antibodies upon vaccination, limited cross-protection has been observed^32–37^. To focus the immune response on stem-specific antibody induction, stem-only HA proteins have been trialled^34,37–39^. Good progress has been reported, with multiple vaccines capable of inducing these stem-directed antibodies subsequently providing a degree of cross-protection^34,36–38,40–45^. However, reduced immunogenicity and the need for improved structure-based design to improve the stability of stem-only constructs, are still hurdles to be overcome.

An alternative approach is the use of platform technologies, which are capable of being rapidly applied to vaccines against a wide range of viral pathogens. Such technologies have proven invaluable in response to the SARS-CoV-2 pandemic in 2020, with numerous vaccines progressing to clinical trials within a matter of months from virus discovery^46–48^. One such technology is the molecular clamp, which utilises the highly stable trimerization domain comprising the 6-helical bundle formed in the post-fusion form of viral fusion proteins, to both constrain trimeric virus glycoproteins in their pre-fusion conformation and serve as a universal purification tag. We have applied this approach to a number of potential emerging viruses^49^, and to the rapid response to the SARS-CoV-2 pandemic^50^. Here, we investigated the application of this platform technology to develop an influenza A virus HA-based vaccine without the limitations of traditional influenza virus vaccine approaches.

## Results

### Characterisation of HA-clamp proteins

**Fig. 1:**
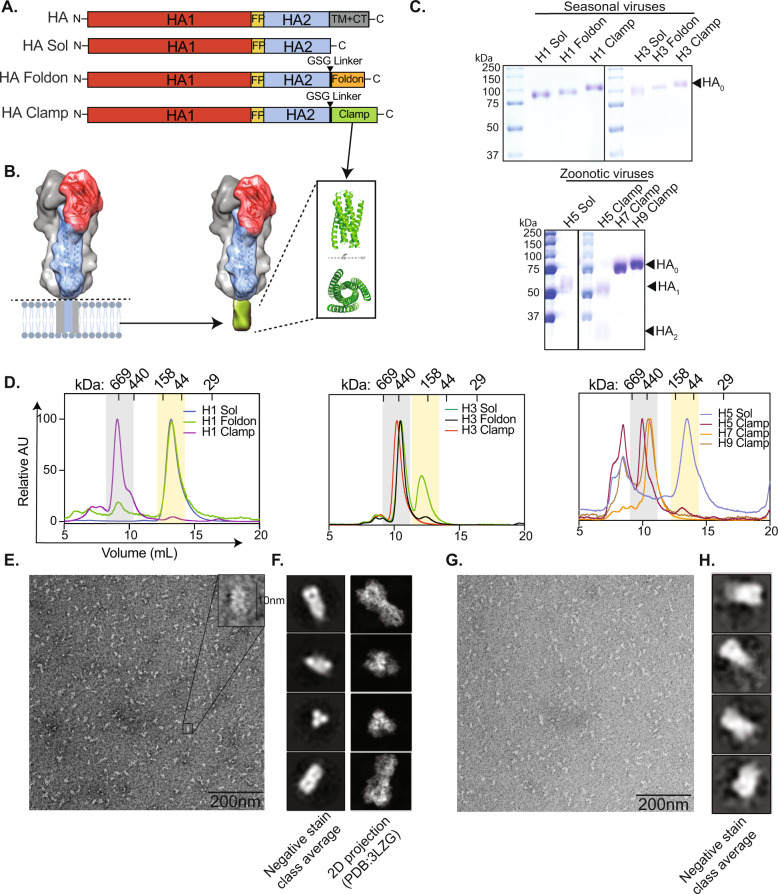
In vitro characterisation of HA clamp antigens. **A** A schematic of the HA gene showing the design of HA, HA ectodomain with no trimerisation domain (HA Sol), HA with the foldon trimerisation domain (HA foldon) and HA with the molecular clamp trimerisation domain (HA clamp). FP fusion peptide, TM transmembrane domain, CT cytoplasmic domain. HA1 is shown in red and HA2 is shown in blue. **B** An illustration of the HA protein with the molecular clamp replacing the transmembrane domain. The structure of the six-helix bundle molecular clamp (PDB ID 3P30) is also illustrated. **C** Coomassie blue stained SDS-PAGE analysis of purified HAs from seasonal and zoonotic influenza A viruses. Molecular weight standards are indicated in kDa. **D** Oligomeric state analysis of HA proteins via size-exclusion chromatography on a Superdex 200 Increase 10/300 GL column. The molecular weight of the standards in kDa is shown. Grey boxes highlight trimeric HA and yellow boxes highlight monomeric HA. **E** Representative micrograph from negative-stain TEM of H1 clamp. **F** 2D class averages of H1 clamp and a 2D reprojection of a representative H1 protein (PDB ID 3LZG). **G** Representative micrograph from negative-stain TEM of H3 clamp and **H** 2D class averages of H3 clamp.

To demonstrate the utility of the molecular clamp stabilisation domain for a recombinant HA-based vaccine, we made several HA constructs from diverse, medically relevant influenza A viruses. HA ectodomains (without the transmembrane or cytoplasmic domains) from recently circulating seasonal H1N1 and H3N2 viruses, as well as those from H5, H7 and H9 avian influenza viruses that have previously caused human infections, were cloned into a mammalian expression vector upstream and in frame with the molecular clamp sequence (HA clamp) separated by a GSG linker. As controls, the HA ectodomain was also cloned into an expression vector with no molecular clamp sequence (termed ‘HA Sol’—soluble) or into a vector containing the foldon trimerisation domain (HA foldon), which has previously been used to stabilise HA^33,51^ (Fig. 1A, B). Plasmids were then expressed using the ExpiCHO expression system and HAs purified from culture supernatant via immunoaffinity purification. Purified proteins were analysed by SDS-PAGE (Fig. 1C), which showed that all proteins were pure and expressed as the uncleaved precursor HA_0_, except for H5 Sol and H5 clamp, which were fully cleaved into the HA_1_ and HA_2_ subunits. This is in line with expectations, as the HA_1_/HA_2_ cleavage site for H5 is polybasic, containing six sequential Arg/Lys amino acids.

Next, proteins were analysed for their oligomeric state via size-exclusion chromatography using a Superdex 200 Increase column. The prefusion conformation of HA exists as a homotrimer^52,53^ and, consistent with this, all HA clamp proteins eluted at a volume consistent with that of a HA trimer, with only minor evidence of aggregation observed (Fig. 1D). Conversely, HA sol proteins were mostly high molecular weight aggregates or monomers, with only H3 sol showing some degree of trimerisation. While the H1 foldon protein separated primarily as monomeric HA with a small proportion of HA trimer, the H3 foldon protein was primarily HA trimer with a minor proportion of monomeric HA. Stabilisation of the soluble trimeric protein was also demonstrated with H5, H7 and H9 (Fig. 1D).

To structurally characterise the H1 and H3 clamp proteins, the peak size-exclusion fractions were collected and analysed by negative-stain transmission electron microscopy (Fig. 1E–H). Proteins appeared homogenous and of the correct size (~10 nm). Additionally, 2D class averages were consistent with reprojections from previously published crystal structures of HA^54^, suggesting that the molecular clamp was indeed constraining HA in its pre-fusion conformation.

Binding of a panel of previously reported mAbs, known to afford protection via neutralisation or Fc effector functions, was also assessed. Both head- and stem-reactive mAbs were chosen and, their binding sites are shown in Supplementary Fig. 1. Head-specific HA mAbs are potently neutralising and protective and tend to be reactive with HA in both its pre- and post-fusion conformations^55–57^. In contrast, many stem-specific mAbs are reactive only with pre-fusion HA owing to the significant structural rearrangements and loss of epitopes that occurs in the HA stem domain upon transition to the post-fusion form. Both head and stem-specific mAb binding affinities were determined by ELISA, with the K_d_ (the antibody concentration at which 50% of the binding response was observed) used to compare relative affinities. The affinities of each mAb tested for the three recombinant HAs are shown in Table 1, while the binding curves and antigen binding sites shown in Supplementary Fig. 1. For H1, H3 and H5 HAs, all proteins, were bound with high apparent affinity by the head-specific mAbs 5J8, C05 and 100F4, respectively (*K*_d_ values from 0.04–1 nM), irrespective of the presence or absence of any stabilisation motif. In contrast, stem-specific mAbs CR6261, CR8043 and FI6v3 showed consistently better recognition of clamp (H1, H3 and H5) and foldon (H1 and H3) constrained HA proteins compared to their HA Sol counterparts, with no detectable binding of either FI6v3 or CR8043 to H3 Sol. While the stem-specific mAbs bound the H1 and H5 Sol proteins, they did so with an apparent affinity 2–8 times lower than for their respective clamp constrained proteins. For the H3 constrained proteins, comparable binding was seen by all mAbs tested except for CR8043, with the H3 clamp showing a ~10 fold increase in binding compared to H3 foldon (*K*_d_ 0.19 nM compared to 2 nM). The H7 and H9 clamp proteins were bound with high apparent affinity by FI6v3 (K_d_ 1.8–2.5 nM).

**Table 1:** ELISA *K*_d_ values.

	*K*_d_ ± SEM (nM)		
	5J8 (head)	CR6261 (stem)	FI6v3 (stem)
H1 Sol	0.33 ± 0.01	0.20 ± 0.04	0.21 ± 0.01
H1 foldon	0.27 ± 0.03	0.045 ± 0.004	0.10 ± 0.01
H1 clamp	0.29 ± 0.02	0.061 ± 0.005	0.10 ± 0.01
	C05 (head)	CR8043 (stem)	FI6v3 (stem)
H3 Sol	0.047 ± 0.004	>300	>300
H3 foldon	0.071 ± 0.005	2.04 ± 0.06	1.21 ± 0.27
H3 clamp	0.039 ± 0.003	0.19 ± 0.03	1.12 ± 0.07
	***K***_**d**_ ± **SEM (nM)**		
	**100F4 (head)**	**CR6261 (stem)**	**FI6v3 (stem)**
H5 Sol	1.05 ± 0.15	16.9 ± 7.8	NT
H5 clamp	1.10 ± 0.23	2.1 ± 0.6	14.5 ± 2.5
	K_d_ ± SEM (nM)		
	FI6v3 (stem)		
H7 clamp	2.5 ± 0.4		
H9 clamp	1.8 ± 0.3		

### Vaccination with HA clamp vaccines elicits potent neutralising antibodies and protection in mice

**Fig. 2:**
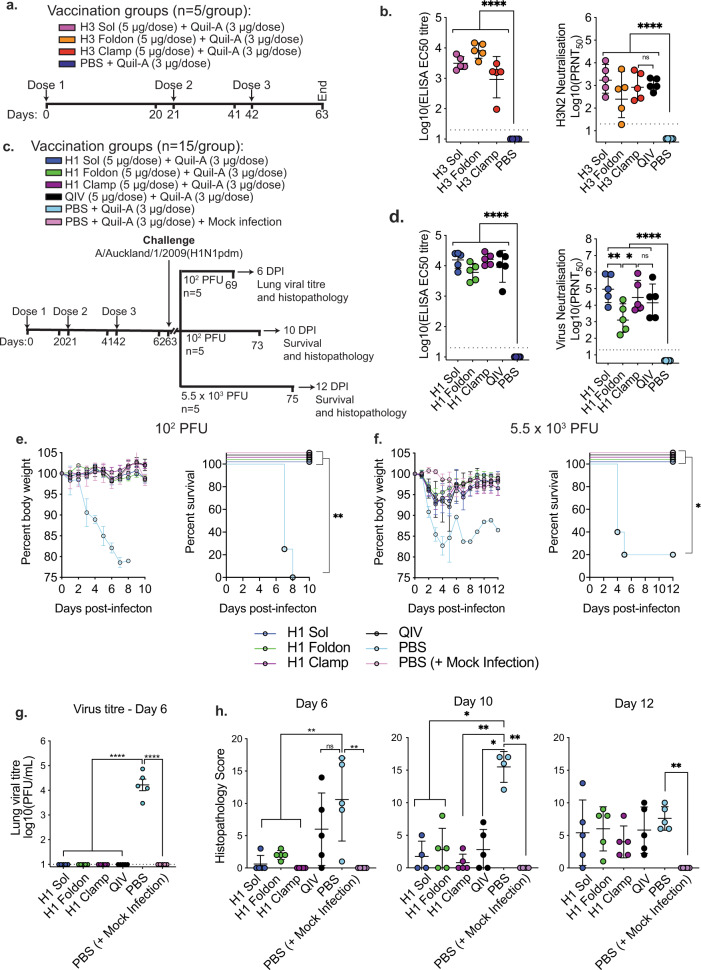
Vaccination with HA clamp induces a neutralising and protective immune response in mice. **A** Schematic of vaccination groups, as well as the bleed and immunisation schedule for the H3 HA vaccination study. **B** Immunogenicity of the H3 vaccine antigens measured by ELISA and PRNT with serum collected on day 63. **C** Schematic of the vaccination groups for the H1 HA challenge study. **D** Immunogenicity of the H1 vaccine antigens measured by ELISA and PRNT from serum collected on day 62. Weight loss and survival curves of mice vaccinated with H1 HA proteins and challenged with **E** 10^2^ PFU and **F** 5.5 × 10^3^ PFU of A/Auckland/1/2009(H1N1pdm09) virus. **G** Virus titre in lung homogenates from mice infected with 10^2^ PFU of virus at day 6 post infection. **H** Histopathology of mice infected with either 10^2^ PFU (day 6 and 10) or 5.5 × 10^3^ PFU (day 12). Data is presented as geometric mean with error bars representing SD. *p < 0.05, **p < 0.005, ***p < 0.0005, ****p < 0.0001, determined using one-way ANOVA with Tukey’s multiple comparison post hoc test.

After confirming that HA clamp proteins could be detected in their pre-fusion, trimeric conformation, we assessed their effectiveness as vaccine candidates alongside HA Sol and HA foldon proteins. C57BL/6 mice (*n* = 5/group) were immunised three times, three weeks apart, with 5 µg of H3 sol, H3 foldon or H3 clamp proteins (from the A/Switzerland/9715293/2013 virus), or with PBS alone, adjuvanted with the saponin-based adjuvant Quil-A (InvivoGen) (Fig. 2A). A commercial quadrivalent inactivated vaccine (QIV, 2015 southern hemisphere formulation, Sanofi) was included as a positive control and was administered with matched adjuvant. This vaccine contained antigen from A/California/7/2009 (H1N1pdm09), A/Switzerland/9715293/2013 (H3N2), B/Phuket/3073/2013 and B/Brisbane/60/2008 viruses. Serum was collected three weeks after the final immunisation and analysed for elicited antibodies, via ELISA, against the cognate antigen. Serum obtained from animals immunised with H3 sol, H3 foldon or H3 clamp all contained high levels of serum IgG (Fig. 2B, left panel). Moreover, when tested for neutralising activity against the matched H3N2 strain (A/Switzerland/9715293/2013) (Fig. 2B), QIV and each of the H3 immunogens induced high titres of neutralising antibodies compared to PBS-immunised controls, although these were not statistically different across groups regardless of the absence or type of trimerisation domain.

Next, recombinant H1 proteins from the A/California/7/2009(H1N1pdm09) virus were assessed for immunogenicity and their ability to induce protection in the mouse model of influenza infection. Mice (*n* = 15/group) were immunised three times, three weeks apart, with H1 sol, foldon or clamp proteins, or with QIV as a positive control, adjuvanted with Quil-A as before. Three weeks after the third dose, mice were bled, then separated into groups of 5 and two groups were challenged intranasally with 10^2^ PFU and one group with 5.5 × 10^3^ PFU of A/Auckland/1/2009(H1N1pdm09) virus. Of the three groups, mice were culled at either day 6 post infection for analysis of the lung viral titre and histopathology of the tissue (one of the 10^2^ PFU groups), or day 10 or 12 post infection (the remaining 10^2^ PFU group and the 5.5 × 10^3^ PFU group) for analysis of recovery from infection. The vaccination and infection regime is outlined in Fig. 2C.

Serum from 5 randomly selected animals, collected prior to infection, was tested for antibody levels and virus neutralisation (Fig. 2D). Again, HA sol, HA foldon and HA clamp were similarly immunogenic and induced neutralising serum antibodies. However, while all three H1 immunogens induced similar levels of HA-specific serum IgG, the neutralising titres induced by H1 foldon immunisation were significantly lower compared to H1 sol and H1 clamp (*p* = 0.0027 and *p* = 0.0494, respectively). A similar trend was noted for the H3 foldon immunogen, although this was not significant (Fig. 2B). All mice immunised with H1 sol, H1 foldon, H1 clamp and QIV were completely protected from both 10^2^ and 5.5 × 10^3^ PFU virus challenge, with no weight loss or mortality observed in any vaccinated groups (Fig. 2E, F). All mice immunised with PBS alone were euthanised due to weight loss following challenge with 10^2^ PFU, and all but 1 mouse was euthanised due to weight loss following challenge with 5.5 × 10^3^ PFU of virus.

**Fig. 3:**
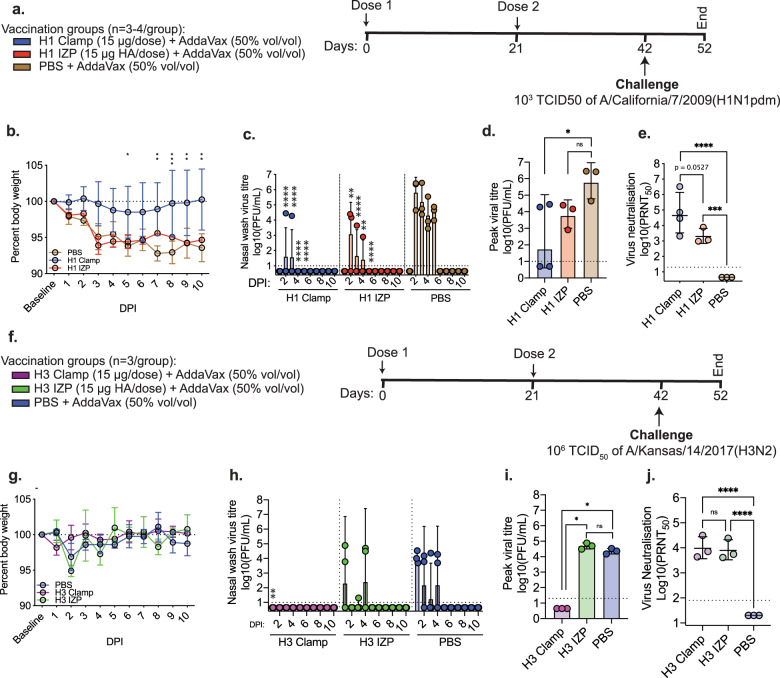
HA clamp provides protection from influenza A virus infection in ferrets. **A** Schematic outlining the vaccination and dose schedule for the H1 ferret study. **B** Weight loss curves, **C** titres of infectious virus recovered from daily nasal washes, **D** peak viral titre in nasal wash samples and **E** titres of neutralising antibody in the serum of vaccinated ferrets prior to virus challenge. **F** Schematic outlining the vaccination and dose schedule for the H3 ferret study. **G** Weight loss curves, **H** titres of infectious virus recovered from daily nasal washes, **I** peak nasal wash viral titre and **J** titres of neutralising antibody in the serum of vaccinated ferrets prior to virus challenge. Bars represent the geometric mean with error bars representing SD while circles show results for individual animals. For virus neutralisation and peak viral titre graphs: ***p < 0.0005, ****p < 0.0001, determined using one-way ANOVA with Tukey’s multiple comparison post hoc test. For nasal wash viral titre graphs: *p < 0.05, **p < 0.005 compared to PBS-immunised animals on the corresponding sampling day, as measured by repeated measures two-way ANOVA with Dunnet’s test for multiple comparisons.

To further investigate this protection, lung tissue collected from mice 6 days after infection with 10^2^ PFU of A/Auckland/1/2009(H1N1pdm09) was homogenised and analysed for viral load via immunoplaque assay (Fig. 2G). Infectious virus was not detected in lung homogenates from any of the HA vaccinated groups or from mice vaccinated with QIV whereas high titres of virus were detected in lungs from all PBS-vaccinated animals. Lung histopathology was also assessed at day 6 and 10 (10^2^) or at day 12 (5.5 × 10^3^ PFU) (Fig. 2H, Supplementary Fig 2). At day 6 post infection, H1 clamp-vaccinated mice showed no evidence of histopathological damage to the lungs, whereas H1 sol- and H1 foldon-vaccinated animals showed evidence of tissue damage in some (H1 sol) or all (H1 foldon) animals. All animals vaccinated with recombinant H1 proteins showed significantly lower (*p* < 0.05) histopathological scores compared to PBS-vaccinated mice, while no significant difference was noted between PBS- and QIV-vaccinated mice at this time. Similar trends were observed at day 10 post infection, with H1 clamp-immunised animals showing lower histopathology scores compared to their sol and foldon counterparts, although all groups showed scores that were significantly reduced compared to PBS-vaccinated mice. At day 12 post infection, all groups showed some level of histopathological damage to the lungs, although none were significantly different compared to PBS-vaccinated mice. Representative histopathology images are shown in Supplenentary Fig. 3.

### Vaccination with HA clamp vaccines elicits protection in ferrets

After demonstrating that molecular clamp-stabilised HA vaccines could protect from influenza virus challenge in the mouse model, we next sought to assess protection in the ferret model. Ferrets are widely regarded as the gold-standard small animal model to study aspects of pathogenesis, transmission and immunity to human influenza virus infections as they can be infected with different human viruses without prior adaptation and they develop clinical signs similar to those in humans^58,59^. To more directly compare HA clamp-stabilised and egg-derived vaccines, we used a monovalent inactivated zonal pool (IZP) rather than the quadrivalent vaccine formulation which could skew results due to inclusion of other virus types/subtypes. In this study, the research-grade squalene oil-in-water emulsion AddaVax was used, as an adjuvant. AddaVax is similar in composition to MF59^®^, an adjuvant used in licenced influenza vaccines^60^.

Ferrets were vaccinated with H1 clamp or IZP vaccines before challenge with A/California/7/2009(H1N1pdm09) as per the schematic in Fig. 3A and monitored daily for weight loss and viral load in nasal washes. Ferrets vaccinated with H1 clamp displayed modest weight loss compared to PBS or H1 IZP-vaccinated ferrets, which showed significantly more weight loss at various days post infection (Fig. 3B). The virus titres detected in nasal wash samples from days 2–5 post infection were significantly reduced in H1 clamp and H1 IZP vaccinated animals (*p* < 0.005) compared to animals vaccinated with PBS and the number of days at which virus shedding could be detected was also reduced (Fig. 3C). Furthermore, the peak viral titres recorded throughout the experiment were significantly lower (*p* = 0.0472) in H1 clamp-vaccinated animals compared to the PBS-immunised but not the IZP immunised ferrets (Fig. 3D). There was a trend for increased titres of neutralising antibody titres in serum collected from H1 clamp-immunised animals prior to infection relative to the H1 IZP-vaccinated group although this was not significant (*p* = 0.0527) (Fig. 3E).

A similar study was performed in ferrets immunised with H3 HA vaccines (Fig. 3F). Ferrets were vaccinated with H3 clamp or H3 IZP vaccines prior to challenge with A/Kansas/14/2017(H3N2). In contrast to H1N1pdm09 challenge, no significant weight loss was observed in any groups following infection with the H3N2 virus (Fig. 3G). Virus was detected in the nasal wash samples from PBS- and H3 IZP-vaccinated animals but no virus was detected in any sample from H3 clamp-vaccinated ferrets (Fig. 3H). The peak virus titre observed across the challenge period was equivalent for PBS- and IZP-vaccinated animals but was below detection for the H3 clamp-vaccinated group (*p* = 0.01 compared to PBS-vaccinated animals) (Fig. 3I). The difference in virus titre between H3 clamp and IZP vaccinated animals was unexpected considering that titres of neutralising serum antibody were not significantly different between these groups prior to virus challenge (Fig. 3J).

### Adjuvanted H1 clamp vaccination induces stem-mediated cross-protection in ferrets

**Fig. 4:**
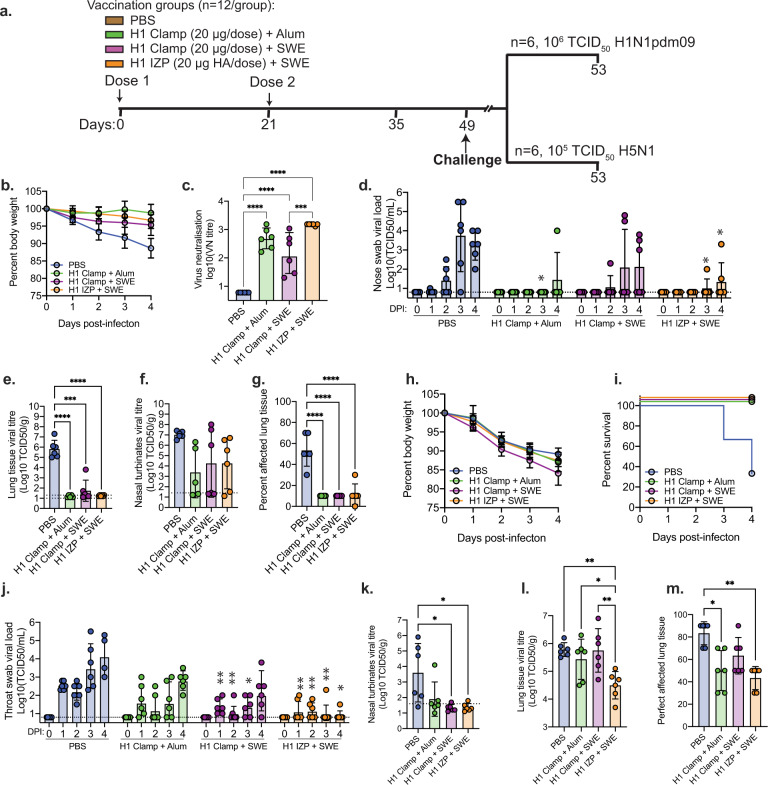
H1 clamp vaccination induces cross-protection in ferrets. **A** Vaccination schedule and dosing outline for the study. **B** Weight loss, **C** virus neutralisation from serum collected on day 49, virus titres detected in **D** nose swabs, **E** lung tissue and **F** nasal turbinates and **G** histology evaluation of lung tissue from ferrets challenged intranasally with the H1N1pdm09 virus (A/Netherlands/602/2009). **H** Weight loss, **I** survival, **J** virus titres in throat swabs, **K** viral load in nasal turbinates, **L** viral load in lung tissue and **M** histology evaluation of lung tissue from ferrets challenged intratracheally with the H5N1 virus (A/Indonesia/5/2005). Data is representative of geometric mean with error bars representing the SD. For virus neutralisation and ELISA graphs: ***p < 0.0005, ****p < 0.0001, determined using one-way ANOVA with Tukey’s multiple comparison post hoc test. For throat and nose swab viral titre graphs: *p < 0.05, **p < 0.005 compared to PBS-immunised animals on the corresponding sampling day, as measured by repeated measures two-way ANOVA with Dunnet’s test for multiple comparisons.

To examine the potential for HA clamp vaccines to elicit heterologous protection, we tested the ability of H1 clamp immunisation to provide protection to ferrets from a highly pathogenic avian influenza (HPAI) virus H5N1 challenge. Ferrets (*n* = 12/group) were immunised with H1 clamp or H1 IZP as a control, with either aluminium hydroxide (here refered to as Alum) or SWE adjuvant, a squalene oil-in-water emulsion available at GMP-grade (Sepivac SWE™), as per Fig. 4A. On the day of the challenge, groups were split evenly and challenged with either a matched-strain H1N1pdm09 virus (via intranasal inoculation) or an HPAI H5N1 virus (via intratracheal inoculation). Following challenge with H1N1pdm09 virus, all animals survived the infection (data not shown) and all vaccines showed a modest effect in reducing weight loss although this did not reach statistical significance (Fig. 4B). Prior to virus challenge, all vaccines induced comparable virus neutralising titres except for H1 clamp + SWE, which induced significantly lower titres compared to H1 IZP + SWE (*p* = 0.001) (Fig. 4C). No serum from H1-immunised ferrets was able to neutralise the H5N1 challenge virus (data not shown). Following virus challenge, daily nose swabs revealed delayed virus detection in all groups when compared to the PBS group (Fig. 4D). This reached statistical significance relative to the PBS group on day 3 for H1 clamp + Alum (*p* = 0.034) and days 3 (*p* = 0.0418) and 4 (*p* = 0.015) for H1 IZP + SWE. In the lung tissue harvested after culling, H1 clamp + Alum and H1 IZP + SWE groups did not have detectable replicating virus measured by TCID_50_ (*p* < 0.0001 compared to the PBS group), and although virus was detected in the H1 clamp + SWE ferrets, it was significantly reduced (*p* = 0.0001) compared to the PBS group (Fig. 4E). In the nasal turbinates, virus was detected in all groups with a trend towards lower levels in vaccinated groups; however, this did not reach statistical significance (Fig. 4F). Furthermore, significantly lower levels (*p* < 0.0001) of affected lung tissue were observed in all groups compared to the PBS group (Fig. 4G).

Upon H5N1 challenge, all groups showed similar weight loss (Fig. 4H), with 4/6 PBS vaccinated animals succumbing to infection by day 4 (Fig. 4I). Animals from all other groups had not succumbed to infection at this point, however, were sacrificed at this time for sample collection. Virus was present in daily throat swabs measured via TCID_50_, albeit at slightly lower levels in vaccinated groups compared to PBS-immunised animals (Fig. 4J). This reached statistical significance on days 1, 2 and 3 for the H1 clamp + SWE group (*p* values from 0.0014 to 0.0173) and days 1, 2, 3 and 4 for the H1 IZP + SWE group (*p* values from 0.0038 to 0.0208). Measurement of viral load in nasal turbinates after culling revealed significantly lower viral loads in H1 clamp + SWE and H1 IZP + SWE groups compared to the PBS group (*p* = 0.029 and *p* = 0.032, respectively) (Fig. 4K). In the lung tissue, however, only H1 IZP + SWE vaccination resulted in significantly lower viral load relative to PBS (*p* = 0.004) (Fig. 4L). This difference also reached statistical significance compared to H1 clamp + Alum (*p* = 0.047) and H1 clamp + SWE (*p* = 0.004). This viral load also translated to a slightly lower percentage of lung tissue pathology; however, this was not statistically significant (Fig. 4M). Thus, while H1 clamp immunisation appears to have some effect at reducing viral load in the nasal turbinates and prolonging death due to infection, there is no difference in weight loss or viral titre.

**Fig. 5:**
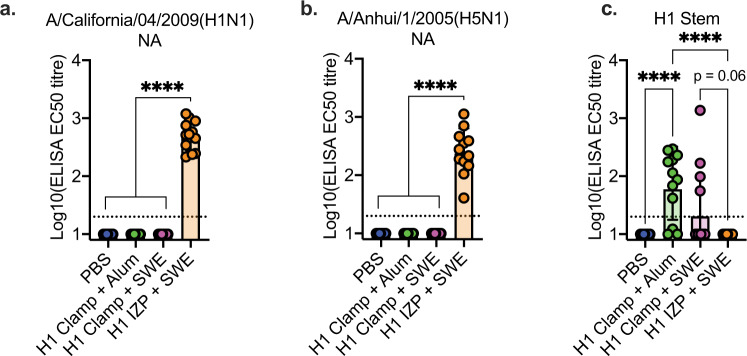
Vaccination with H1 IZP or H1 clamp induces NA and stem-specific antibodies, respectively. Serum from ferrets vaccinated with 2 doses of either PBS, H1 clamp adjuvanted with either Alum or SWE, or H1 IZP adjuvanted with SWE was collected 4 weeks after the second dose and assessed for binding to **A** neuraminidase (NA) from a H1N1pdm09 virus, **B** NA from a H5N1 virus and **C** the stem domain of H1 HA. Data is representative of geometric mean with error bars representing the SD. ***p < 0.0005, ****p < 0.0001, determined using one-way ANOVA with Tukey’s multiple comparison post hoc test.

We hypothesised that any cross-protection from a H1 vaccine against a H5N1 virus challenge would either be due to antibodies against conserved HA stem domain epitopes or antibodies directed against any residual NA present in the IZP vaccine—two phenomena that have been previously observed in the context of influenza A virus cross-protection^61,62^. To investigate this further, serum collected prior to challenge was assayed for binding to H1N1 or H5N1 NA (Sino Biological), or an in-house expressed H1 stem construct based on previously designed antigens^37^. Only serum from H1 IZP-vaccinated ferrets contained anti-NA antibodies (Fig. 5A, B) whereas only serum from H1 clamp-vaccinated ferrets contained anti-H1 stem antibodies, with titres being highest when adjuvanted with Alum (Fig. 5C).

## Discussion

The risk of a pandemic influenza virus emerging remains high and, until a truly universal vaccine is available, rapid development of a vaccine targeting an emerging pandemic strain is the only option. Egg-based production of vaccines against pandemic strains has been used successfully in the past, most notably in 2009^63^; however, scale and timelines remain challenging. Platform technologies, such as those utilised to respond to the SARS-CoV-2 pandemic, offer unique advantages^46–48^. In this study, we demonstrate that the ‘molecular clamp’ constrains the HA proteins from medically relevant influenza A viruses of different subtypes (H1, H3. H5, H7 and H9) to their pre-fusion, trimeric conformation, and induces a potent protective immune response.

Compared to non-stabilised or foldon-stabilised counterparts, the molecular clamp was associated with enhanced trimerization of HA, with little dissociation into monomeric HA observed. It has been widely reported that stabilisation of recombinant HA with either foldon or GCN4 trimerisation domains is sufficient for the expression of correctly folded pre-fusion protein^33,35^. However, here we found that this may not be applicable to every HA strain, given our observation that H1 foldon dissociated into mostly monomeric HA, with only a small amount of trimeric HA present. In contrast, SEC analysis and pre-fusion specific mAb binding showed that all molecular clamp-stabilised HAs were purified as trimeric, pre-fusion HA. This improved trimerisation profile when compared to foldon is likely due to the higher thermal stability of the clamp domain^64,65^.

Improved trimerisation and pre-fusion folding of clamp-stabilised HA correlated with enhanced protection in mouse and ferret models of matched strain infleunza A infection. All vaccine candidates provided protection against homologous virus challenge, with HA clamp providing a trend towards lower virus-induced lung pathology in mice. In ferrets, peak viral loads were reduced in nasal wash samples from HA clamp-immunised animals compared to their IZP counterparts for ferrets challenged with both H1N1pdm09 and H3N2 viruses. Furthermore H1pdm clamp provide significant level of protection against weight loss relative to the matched IZP. Immunising with HA that is correctly folded into the pre-fusion conformation, mimicking that found on the infectious virion, would be expected to induce antibody responses that are better able to recognise epitopes expressed by native HA on infectious virions and mediate protection. Interestingly, our studies demonstrated that titres of neutralising antibodies induced by stabilised and non-stabilised HA were roughly equivalent. This finding is in contrast to studies using respiratory syncytial virus (RSV), where antibodies specific to the pre-fusion confirmation of the viral F glycoprotein were responsible for the majority of neutralisation activity^66^. However, our results with influenza A virus are perhaps not surprising given that it is well established that the head domain of HA is the primary target of neutralising antibody responses^67^ and this domain was shown to remain intact in non-stabilised HA. Despite equivalent titres in serum neutralisation responses, constraining HA in its trimeric pre-fusion conformation could also improve protective efficacy by stimulating antibodies to non-neutralising epitopes, particularly those directed towards the stem domain.

Vaccination of ferrets with adjuvanted H1 clamp also provided a limited level of protection from a divergent H5N1 virus challenge, demonstrating a level of cross-protective efficacy for clamp-stabilised HA vaccines. While no differences in weight loss were seen between groups, H1 clamp-immunised animals showed delayed time to death and differences in viral load from daily throat swabs. This protection was likely due to induction of stem-specific antibodies in the H1 clamp + Alum group. Such antibodies have previously been shown capable of protecting via Fc-mediated effector functions^61,68–70^. Non-stem specific antibodies and/or cellular immune responses, both of which warrant further investigation, are also likely contributing to this protection.

While cross-protection against H5N1 challenge after H1-based vaccination has been observed previously, these are from stem-only HA vaccine candidates^34,36,37^, which lack the head domain and therefore cannot induce potent neutralising head-specific antibodies^27,28,55,56,71–74^. In contrast, the H1 clamp vaccine induces strain-specific neutralising antibodies directed towards the head domain, as well as stem-specific antibodies capable of providing a small level of cross-protection. While stem-only antibodies have been shown to be protective, studies have also shown them to be present at low levels of ~0.1 µg/mL in human serum and are only boosted ~2-fold after vaccination with an inactivated vaccine^75^. In order to provide protection, it has been suggested that these levels would need to be boosted to ~20 µg/mL, or over 20-fold^72^. Conversely, head-specific antibodies appear to be induced to much higher levels and can also potently neutralise the virus. Thus, HA clamp immunisation, which elicits both head- and stem-specific antibody responses, could provide an advantage in the context of influenza virus protection by inducing head-specific antibodies at high levels to provide neutralisation, albeit with a lower breadth against heterologous strains, while concurrently inducing stem-specific antibodies capable of providing protection against heterologous strains.

Cross-protection against the same H5N1 challenge was also induced after vaccination with H1 IZP, in which no stem-specific antibodies were induced. Instead, the H1 IZP vaccination induced NA-specific antibodies (Fig. 5A, B), which have been shown to be able to provide cross-protection between H1N1 and H5N1 in ferrets^62^ and mice^76^. While these distinct mechanisms of cross-protection both proved successful, a combination of the two may prove advantageous against even more divergent viruses in the future. Indeed, efforts are being made to quantify and standardise the NA content of current inactivated vaccines in order to assess the impact of NA-specific antibodies in protection in the human population^77^. Investigation of pre-fusion HA vaccine inducion of stem-specific HA antibodies and recombinant neuraminidase inducing cross-reactive NA antibodies could lead us one step closer to universal protection.

In this work, multiple adjuvants were used, and the HA clamp proteins proved potent and successful as vaccines with each adjuvant. This demonstrates the robustness of the clamp platform for influenza A viruses, however, making comparison of results between experiments more difficult. Therefore, the use of different adjuvants is also worth exploring in more detail. Selecting adjuvants likely to boost IgG responses capable of mediating antibody-dependent cellular cytotoxicity could improve cross-protection^78^. Our group has explored the use of different adjuvants^79^; however, expanding on this study to include a more diverse adjuvant panel with experiments performed in parallel allowing for head-to-head comparisons would prove valuable. This would also allow more in-depth T-cell analysis in the context of live virus protection and cross-protection in mouse and ferret challenge models, providing valuable insight into the best formulations to achieve the best protection from influenza A virus infection.

This is the first study to demonstrate the molecular clamp technology for influenza A virus vaccine development, offering a promising alternative to traditional influenza vaccine approaches.

## Methods

### Animal ethics statement

All animal experiments were approved by the relevant animal ethics committees at either the University of Queensland (AEC number SBMS/071/17 or the University of Melbourne (AEC number 1714278.4). The ferret experiments carried out at Viroclinics Xplore in Schaijk, The Netherlands, were under conditions that meet the standard of Dutch law for animal experimentation. The facility is fully accredited by the Dutch ministry that governs and inspects the animal facilities and oversees, coordinates and inspects activities of the animal ethics committees of Dutch institutions and academic centres. A registered article 9 officer is responsible for the design and management of the experiments, in close consultation with the animal welfare body (IvD) who grated ethical approval for the experiments, registered under Working Protocol number: AVD277002015142-1-WP21.

### Cells

Madin-Darby Canine Kidney (MDCK) cells were maintained in Dulbecco’s Modified Eagle Medium (DMEM) supplemented with 10% heat-inactivated fetal bovine serum (FBS) with 100 U/mL penicillin and 100 µg/mL streptomycin (Gibco). Chinese hamster ovary cells (ExpiCHO-S, ThermoFisher) were maintained in suspension culture with rotary shaking (125 rpm) in ExpiCHO Expression Medium (ThermoFisher). All mammalian cells were cultured at 37 °C with 5–8% CO_2_.

### Viruses

Virus stocks were kindly provided by the WHO Collaborating Centre for Reference and Research on Influenza, Melbourne, Australia or ViroClinics Biosciences, Rotterdam, The Netherlands. The viruses used in this work were A/Auckland/1/2009 (H1N1pdm09), A/Switzerland/9715293/2013 (H3N2), A/Kansas/14/2017 (H3N2), A/California/7/2009 (H1N1pdm09), A/Netherlands/602/2009(H1N1pdm09) and A/Indonesia/5/2005(H5N1). Viruses were propagated in the allotonic fluid of embryonated hen’s eggs or in MDCK cells, as previously described^80,81^.

### Cloning of HA constructs and antibodies

The cDNA sequence encoding the ectodomain of desired HAs, or the variable domains from the heavy and light chains of the antibodies, were codon optimised for Chinese hamster (*Cricetulus griseus*) expression before synthesis as Geneblocks (Integrated DNA Technologies) together with the necessary 5′ and 3′ overlaps for subsequent InFusion cloning (ClonTech) into in-house generated mammalian expression vectors. These vectors contained either no trimerisation domain, the foldon trimerisation domain or the molecular clamp domain (derived from HIV-1 glycoprotein 41 heptad repeat (HR) regions, HR1 (aa540–576) and HR2 (aa619–656)) for the HA constructs, or the constant regions of human or mouse IgG1 heavy or light chain for the antibodies. The HA stem constructs, which were based on previously designed antigens^37^ were cloned into a vector containing a monomeric Fc domain^82^. After successful InFusion cloning, plasmid DNA was prepared by Midi- or Maxi-prep (Promega) and sequence confirmed at the Australian Genome Research Facility (AGRF).

### Expression and purification of antibodies and HA constructs

Antibodies and recombinant HA proteins were transiently expressed using the ExpiCHO Expression System (ThermoFisher) as per the manufacturer’s instructions. Supernatants were harvested on day 5–7 by centrifugation at 5000 × g for 10 min at 4 °C before filtration using a 0.22 µm filter. Antibodies and Fc-fusion proteins were purified on an AKTApure FPLC via protein A affinity purification using a HiTrap Protein A HP column, as per the manufacturer’s instructions. Recombinant HA proteins were purified via immunoaffinity columns, which were made in-house by coupling HA-specific antibodies to HiTrap NHS-activated HP columns. HA clamp proteins were purified using the clamp-specific antibody HIV1281^83,84^ as previously described^49,79^, with those HA proteins not containing the clamp purified in a similar fashion but using HA-specific mAbs (5J8^55^, C05^56^ or 100F4^57,85^) in place of the clamp-specific mAb. In-house made immunoaffinity columns were equilibrated with phostphate-buffered saline (PBS) pH 7.4, and elution was done with 100 mM Tris, 400 mM NaCl, 5 mM EDTA, 20 mM diethylamine, pH 11.5. Proteins were concentrated using Amicon 30 kDa MWCO centrifugal filter units as per the manufacturer’s instructions. Purity was assessed via SDS-PAGE and oligomeric state assessed via size-exclusion chromatography using a Superdex 200 Increase 10/300 GL column.

### ELISA of HA constructs with monoclonal antibodies or serum

Transiently expressed HA or commercially supplied recombinant NA (Sino Biological) proteins were coated at 2 µg/mL in PBS on Nunc Maxisorp ELISA plates overnight at 4 °C. The next day, plates were blocked with 5% milk diluent blocking concentrate (Seracare) for 30–60 min at room temperature. Plates were then probed with serial dilutions of primary antibodies (either serum or monoclonal antibodies) for 1 h at 37 °C, followed by 3x washes with PBS.T. The relevant HRP-conjugated secondary antibody (goat anti-mouse, goat anti-human or goat anti-ferret, all diluted to 1 in 2000) was then added for 1 h at 37 °C before washing as before. TMB substrate (Life Technologies) was added and reactions stopped after 5–10 min with 1 M H_2_SO_4_ before absorbance was read at 450 nm using a SpectroMax 190 Microplate Reader (Molecular Devices).

### Transmission electron microscopy of HA proteins

To visualise correct folding of HA proteins, peak fractions from size-exclusion chromatography analysis were diluted to ~10 µg/mL in PBS and applied to glow-discharge carbon films supported by formvar on 400-mesh copper grids (ProSciTech). The samples were blotted for 2 min and washed three times with water prior to staining with 1% uranyl acetate. Samples were air dried and then imaged on a Hitachi HT7700 transmission electron microscope at 120 kV and 30k magnification corresponding to a nominal pizel size of 4.4 Å. CTF 2D class averaging was performed using Relion 3.0.

### Adjuvants and vaccine formulation

Quil-A, Alum and AddaVax were purchased commercially (InvivoGen), and SWE squalene oil-in-water emulsion was provided by Seppic (France). Vaccines containing these adjuvants were formulated shortly before injection. Quil-A was made as a stock solution to 10 mg/mL in PBS, and the desired amount was added to the formulation before being made up to the final desired volume (20 µL/dose) using PBS. Alum and AddaVax were mixed volume to volume with vaccine antigens to obtain a final antigen concentration of 100 µg/mL.

Physiochemical properties of the SWE adjuvant and HA clamp antigen integrity were monitered 24 h after the initial mixing of the vaccine formulation components using a sandwich ELISA including 5J8 and FI6v3 anti-HA mAbs. Osmolarity of the final HA Clamp SWE formulations was in the 200–230 mOsm/kg range.

### Mouse immunisation studies

Naive 6–8-week-old female C57BL/6 mice (Animal Resources Centre) were maintained in a pathogen-free environment at the University of Queensland’s Biological Resources animal facility within the Australian Institute of Bioengineering and Nanotechnology. Mice were immunised intradermally (i.d.) with 5 µg of vaccine antigen with 3 µg of Quil-A adjuvant, diluted with PBS to a final volume of 20 µL. When Addavax was used as an adjuvant, 25 µL of Addavax was mixed with 25 µL of PBS containing 5 µg of the vaccine antigen. Vaccines were then administered via the intramuscular route. Blood was taken via tail bleed one day before each vaccination and the day before challenge. Three weeks after the final vaccination, mice were anaesthetised using isofluorane and challenged intranasally with virus diluted in 50 µL of PBS. All mice were monitored daily for weight loss, and when necessary, mice were sacrificed via intraperitoneal injection of Lethobarb (100 µL of a 14 mg/mL solution in saline) and blood harvested via cardiac bleed. The commercial quadrivalent inactivated vaccine used contained antigens from A/California/7/2009(H1N1), A/Switzerland/9715293/2013(H3N2), B/Phuket/3073/2013 and B/Brisbane/60/2008 viruses.

### Histopathology analysis of mouse lung samples

The left lobe of the mouse lung was placed in 1 mL of 4% paraformaldehyde dissolved in PBS. Samples were then embedded in paraffin, sectioned into 4 µm slices and stained with haematoxylin and eosin. Slides were then scored by a sample-blind veterinary pathologist.

### Determination of viral titres in mouse lungs

The superior, middle and inferior lobes of the right lung of the mice were harvested and placed in 1 mL DMEM supplemented with 100 U/mL of penicillin and 100 µg/mL of streptomycin (Gibco). Tubes containing lungs were weighed before addition of a 4 mm diameter grinding ball. Tissue was homogenised using a TissueLyser II (Qiagen) for 4 min at a frequency of 1/25. Samples were centrifuged for 5 min at 10,000 × g before supernatants harvested and aliquoted for storage at −80 °C. Lung homogenate supernatant was titrated in serum-free DMEM supplemented with 2 µg/mL of TPCK-treated trypsin (Sigma Aldrich) before being added to a confluent monolayer of MDCK cells in a 96-well tissue culture plate. Infection was allowed to occur for 1 h at 37 °C before homogenate samples removed and 100 µL/well of overlay medium was added (1.5% carboxymethylcellulose, 2% FBS, 100 U/mL of penicillin and 100 µg/mL of streptomycin). Cells were incubated for 3 days at 37 °C with 5% CO_2_ before overlay was removed and cells fixed with 80% acetone in PBS for 20 min at −20 °C. Virus plaques were then detected via immunoplaque assay as previously described^86,87^ using the HA-specific mAb FI6v3 and a goat anti-human IRDye 800CW secondary antibody (LI-COR). Plates were imaged on an Odyssey infrared imaging and plaques counted by eye to calculate plaque-forming units/mL.

### Ferret challenge studies

Ferrets were randomly assigned into groups of 3–6 and immunised intramuscularly in a dose volume of 0.2 mL under ketamine anaesthesia (20 mg/kg). Two immunisation doses, 2–3 weeks apart were given. Animals were challenged 2–4 weeks after the final immunisation via intranasal or intratracheal inoculation as previously described^88^. Ferrets were monitored daily for temperature and weight loss, and nasal washes or swabs (nasal and throat) were taken for virological analysis while the animals were under ketamine anaesthesia as before. For the study conducted at Viroclinics Xplore, sixty male ferrets, approximately 9 to 10 months old, were randomly assigned into ten groups with six animals per group. Ferrets were immunised, using a prime boost regimen four weeks apart, with a dose volume of 200 µl via the intramuscular route of administration. Three weeks following the boost immunisation, ferrets were challenged with either 10^6.0^ TCID_50_/dose A/Netherlands/602/2009 (H1N1pdm09) virus (*n* = 30) or 10^5.0^ TCID_50_/dose A/Indonesia/5/2005 (H5N1) virus (*n* = 30). Both challenges were administered via the intracheal route using a total dose volume of 3.0 ml. Following challenge, nose and throat swabs were sampled daily and respiratory tissues were collected on day 4 post challenge following euthanasia by abdominal exsanguination.

### Virological analysis of ferret samples

Swabs and homogenised tissue samples were used for the detection of replication competent virus in a TCID_50_ assay. To this end, quadruplicate 10-fold serial dilutions were prepared and incubated on MDCK monolayers. After washing, cell monolayers were incubated for 6 days at 37 °C. Next, culture supernatants were harvested, turkey erythrocytes added and the plates were incubated for 1 h at 4 °C. HA patterns were read and titres were calculated using the method of Spearman–Karber.

### Virus neutralisation assays

Virus neutralisation of serum samples was tested either via plaque reduction neutralisation test (PRNT) as previously described^86,87^ using the HA-specific mAb FI6v3 to visualise plaques, or via TCID_50_ as previously described^88^. Plaques were counted by eye or using the software package Viridot^89^. The IC50 value was determined using GraphPad Prism 8 software using a inhibitor vs response (three parameters) model. All serum was treated with receptor destroying enzyme (RDE[II], Denka Seiken Co.) prior to analysis.

### Reporting summary

Further information on research design is available in the Nature Research Reporting Summary linked to this article.

## Supplementary information

Supplementary Information

Reporting Summary

## Data Availability

The datasets generated during and/or analysed during the current study are available from the corresponding author on reasonable request.
