# Author Correction: Development of molecular clamp stabilized hemagglutinin vaccines for Influenza A viruses

**DOI:** 10.1038/s41541-021-00428-y

**Published:** 2022-01-05

**Authors:** Christopher L. D. McMillan, Stacey T. M. Cheung, Naphak Modhiran, James Barnes, Alberto A. Amarilla, Helle Bielefeldt-Ohmann, Leo Yi Yang Lee, Kate Guilfoyle, Geert van Amerongen, Koert Stittelaar, Virginie Jakob, Celia Lebas, Patrick Reading, Kirsty R. Short, Paul R. Young, Daniel Watterson, Keith J. Chappell

**Affiliations:** 1 School of Chemistry and Molecular Biosciences, The University of Queensland, St Lucia, QLD 4072 Australia; 2 WHO Collaborating Centre for Reference and Research on Influenza, Peter Doherty Institute for Infection and Immunity, Melbourne, VIC 3000 Australia; 3 Australian Infectious Diseases Research Centre, Global Virus Network Centre of Excellence, Brisbane, QLD 4072 and 4029 Australia; 4 School of Veterinary Science, The University of Queensland Gatton Campus, Gatton, QLD 4343 Australia; 5 Viroclinics Xplore, Landerd Campus, Nistelrooise Baan 3, 5374 RE Schaijk, The Netherlands; 6 Vaccine Formulation Institute, Chemin des Aulx 14, 1228 Plan-Les-Ouates, Geneva, Switzerland; 7 Department of Microbiology and Immunology, Peter Doherty Institute for Infection and Immunity, The University of Melbourne, Parkville, VIC 3000 Australia; 8 The Australian Institute of Biotechnology and Nanotechnology, The University of Queensland, St Lucia, QLD 4072 Australia

**Keywords:** Protein vaccines, Influenza virus

Correction to: *npj Vaccines* 10.1038/s41541-021-00395-4, published online 08 November 2021

In this article, the author name Virginie Jakob was incorrectly written as Virginie Jakon. The original article has been corrected.

