# Supplementary material for: Development of molecular clamp stabilized hemagglutinin vaccines for Influenza A viruses
Source: NPJ Vaccines. 2021 Nov 8;6:135. doi: 10.1038/s41541-021-00395-4 (PMC8575991; doi:10.1038/s41541-021-00395-4)
Supplement: Supplementary file 1 — Supplementary Information [file 41541_2021_395_MOESM1_ESM.pdf]

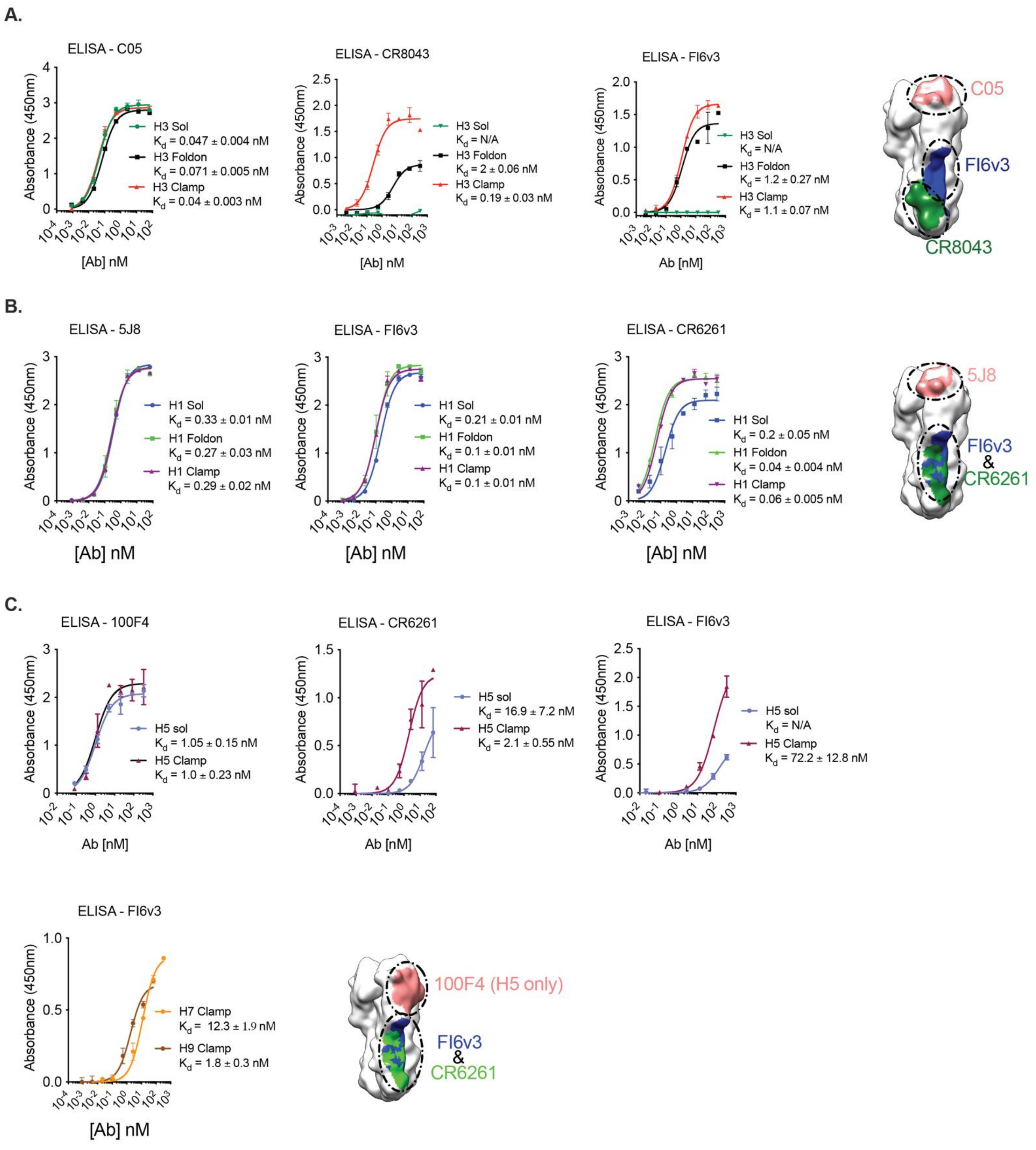

Supplementary figure 1: ELISA curves of mAbs binding to different HA proteins. Recombinant HA proteins from **A.** H3 HAs, **B.** H1 HAs or **C.** Zoonotic HAs were tested for binding by HA-specific monoclonal antibodies via ELISA. The binding sites of each antibody is indicated on the HA structures.

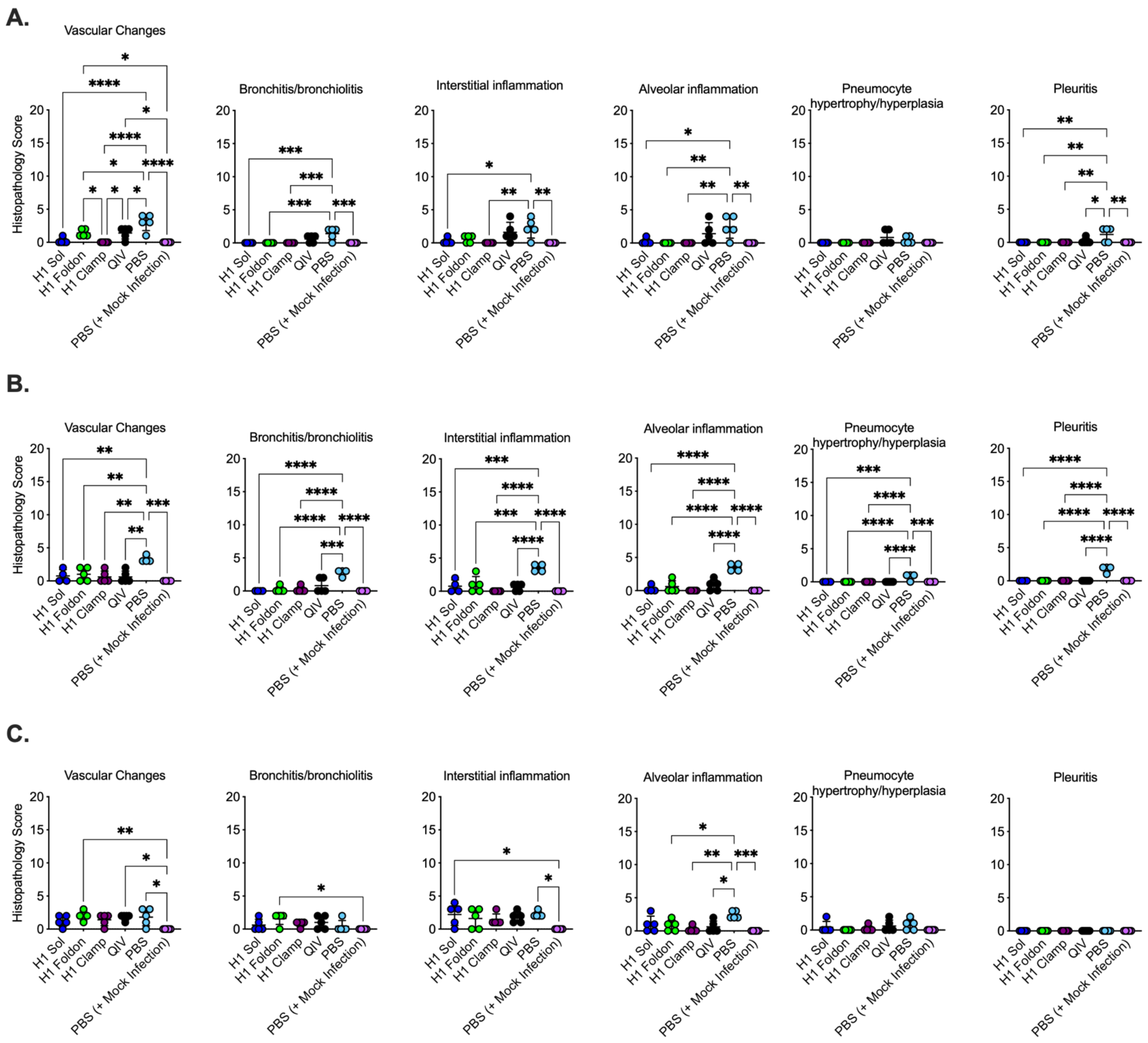

Supplementary figure 2: Histopathology scores from mice challenged with H1N1pdm virus. Mice were vaccinated with H1 HA proteins before challenge with either **A.** 10^2 PFU with lungs harvested on day 6 post-infection, **B.** 10^2 PFU with lungs harvested on day 10 post-infection (or when the animals reached the humane endpoint) or **C.** 5.5 x 10^3 PFU with lungs harvested on day 12 post-infection (or when the animals reached the humane endpoint). * = $p < 0.05$, ** = $p < 0.005$, *** = $p < 0.0005$, **** = $p < 0.0001$, determined using one-way ANOVA with Tukey's multiple comparison *post hoc* test.

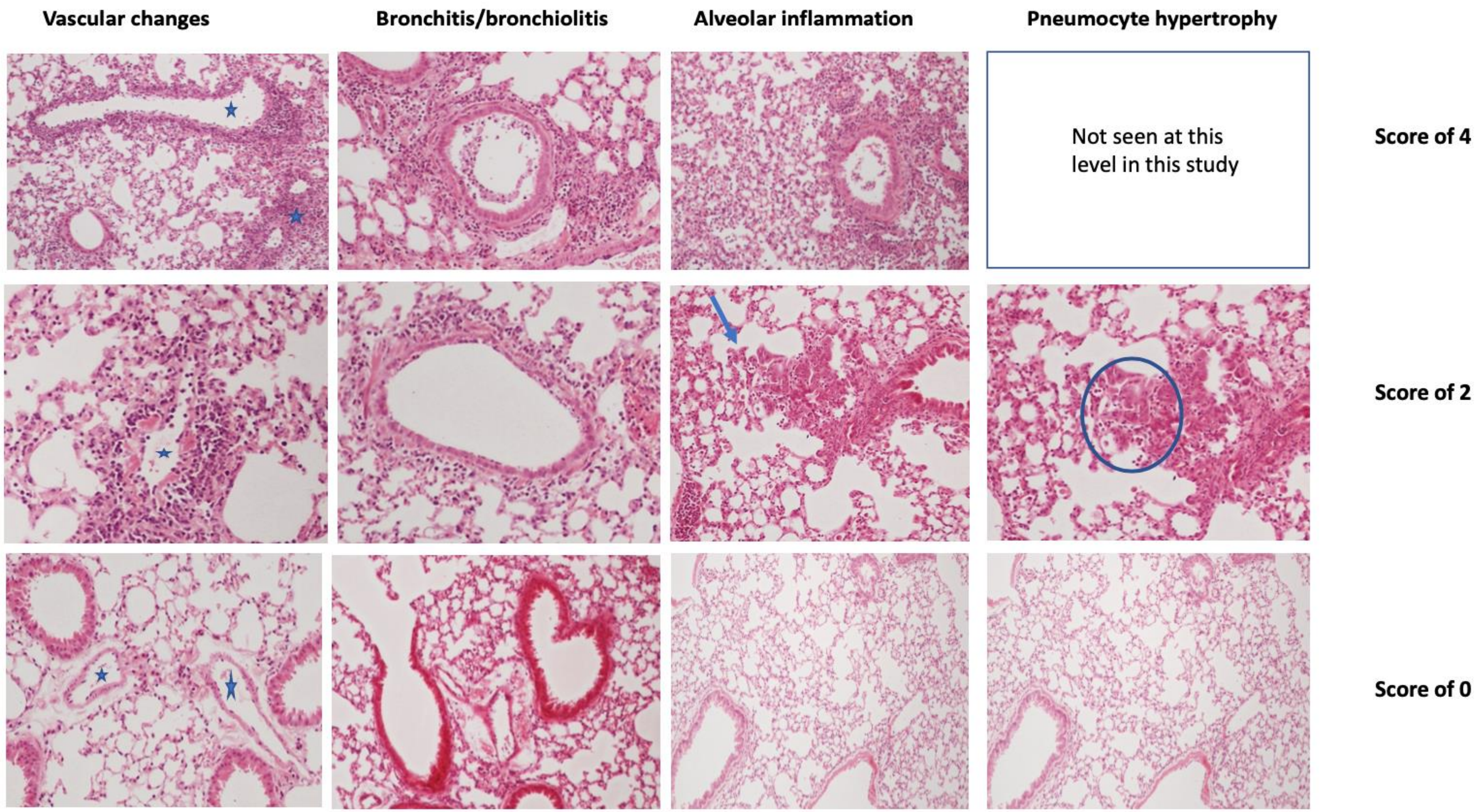

Supplenentary figure 3: Representative microphotos of the types of pathological changes assessed and the degree of severety, from within normal limits (score of 0) to mild (score of 2) to severe (score of 4). Scores of 1 (subtle/minimal change) and of 3 (moderate change) not shown. Stars indicate pulmonary vessels. The arrow in the right-centre panel indicates an area of mild alveolitis with focal pneumocyte hypertrophy , show in the enlarged microphoto on the right (circled). Hematoxylin & eosin stained sections taken at 40x or 100x magnification.

Supplementary table 1: Sequences of proteins used in this manuscript.

Igκ signal sequence is in italics, and the Foldon and 6HB domains in bold.

**Virus strain – protein name:**	**Sequence:**	
A/Switzerland/9715293/2013(H3N2) - H3 Sol	DNA	ATGAAGACCATCATCGCTCTGTCATACATCCTTTGTCTGGTGTTTGCCCAGAAGCTGCCAGGTAATGATAACTCAACAGCTACTCTTTGTCTGGGTCATCATGCAGTGCCCAACGGCACCATTGTCAAGAC<br>CATCACCAACGATCGGATAGAAGTGACCAACGCCACCGAATTGGTGCAGAACAGCTCAATCGGAGAGATCTGCGATAGTCCTCATCAAATTTTGGACGGAGAAAACTGCACCTTGATAGATGCTTTGCT<br>GGGCGATCCTCAGTGCGACGGCTTCCAAAACAAGAAGTGGGACCTTTTTGTGGAGCGCTCTAAGGCCTATTCCAATTGCTATCCATACGATGTTCCAGATTATGCCTCCCTGCGGTCACTTGTCGCTAGTA<br>GCGGAACACTGGAGTTTAACAACGAGAGTTTCAATTGGGCTGGAGTGACCCAGAACGGCACCAGTTCTTCATGCAGACGAGGGTCAAATTCTTCATTTTTCTCTAGGCTCAACTGGCTCACTCATCTCAA<br>TAGTAAATACCCAGCTCTTAACGTGACTATGCCTAACAATGAGCAATTCGACAAGTTGTACATCTGGGGTGTGCACCATCCTGTCACAGATAAAGATCAGATCTTTCTTTACGCCCAGTCCTCCGGCCGTA<br>TAACAGTGTCAACTAAGCGCAGTCAGCAGGCTGTGATCCCTAACATCGGATACAGACCTAGGATACGCGACATCCCAAGCCGCATCTCCATCTACTGGACTATTGTAAAACCTGGCGACATCCTTCTGAT<br>CAACAGCACCGGCAACCTCATCGCTCCTCGCGGGTATTTTAAGATCCGCTCCGGCAAAAGTAGCATTATGAGGTCCGACGCACCTATCGGCAAGTGCAAGTCAGAGTGTATTACACCAAACGGAAGCAT<br>TCCCAATGACAAACCATTCCAGAACGTGAACCGCATCACCTATGGCGCCTGTCCAAGATACGTGAAGCAGTCTACCCTGAAACTGGCCACAGGTATGCGGAACGTGCCCGAGAGGCAAACCCGAGGCAT<br>CTTCGGGGCAATCGCAGGCTTCATCGAGAACGGTTGGGAAGGCATGGTCGATGGCTGGTACGGATTTAGACATCAGAACAGTGAAGGGAGAGGCCAAGCCGCTGATTTGAAGTCCACCCAGGCAGCCA<br>TCGACCAGATTAACGGCAAGCTGAACAGGTTGATAGGGAAAACAAATGAAAAGTTTCATCAAATTGAGAAGGAGTTTTCTGAGGTTGAGGGCAGGATTCAGGATCTGGAAAAGTATGTGGAAGATACT<br>AAGATCGATCTGTGGAGCTATAACGCCGAGCTGCTGGTAGCCCTGGAGAATCAGCACACAATTGACCTCACAGATTCTGAGATGAACAAACTGTTCGAAAAGACTAAGAAGCAACTCAGGGAAAATGC<br>AGAAGATATGGGCAATGGCTGTTTCAAAATCTATCATAAGTGTGACAATGCTTGTATCGGTTCCATCAGGAACGGCACTTATGACCATGACGTCTACAGGGATGAAGCCCTGAATAACCGGTTTCAGATC<br>AAGGGAGTCGAACTGAAGAGCGGATACAAAGAT
	Amino acid	MKTIIALSYILCLVFAQKLPGNDNSTATLCLGHHAVPNGTIVKTITNDRIEVTNATELVQNSSIGEICDSPHQILDGENCTLIDALLGDPQCDGFQNKKWDLFVERSKAYSNCYPYDVPDYASLRSLVASSGTLEFNN<br>ESFNWAGVTQNGTSSSCRRGSNSSFFSRLNWLTHLNSKYPALNVTMPNNEQFDKLYIWGVHHPVTDKDQIFLYAQSSGRITVSTKRSQQAVIPNIGYRPRIRDIPSRISIYWTIVKPGDILLINSTGNLIAPRGYFKIRS<br>GKSSIMRSDAPIGKCKSECITPNGSIPNDKPFQNVNRITYGACPRYVKQSTLKLATGMRNVPERQTRGIFGAIAGFIENGWEGMVDGWYGFRHQNSEGRGQAADLKSTQAAIDQINGKLNRLIGKTNEKFHQIEKE<br>FSEVEGRIQDLEKYVEDTKIDLWSYNAELLVALENQHTIDLTDSEMNKLFEKTKKQLRENAEDMGNGCFKIYHKCDNACIGSIRNGTYDHDVYRDEALNNRFQIKGVELKSGYKDW*
A/Switzerland/9715293/2013(H3N2) - H3 Foldon	DNA	ATGAAGACCATCATCGCTCTGTCATACATCCTTTGTCTGGTGTTTGCCCAGAAGCTGCCAGGTAATGATAACTCAACAGCTACTCTTTGTCTGGGTCATCATGCAGTGCCCAACGGCACCATTGTCAAGAC<br>CATCACCAACGATCGGATAGAAGTGACCAACGCCACCGAATTGGTGCAGAACAGCTCAATCGGAGAGATCTGCGATAGTCCTCATCAAATTTTGGACGGAGAAAACTGCACCTTGATAGATGCTTTGCT<br>GGGCGATCCTCAGTGCGACGGCTTCCAAAACAAGAAGTGGGACCTTTTTGTGGAGCGCTCTAAGGCCTATTCCAATTGCTATCCATACGATGTTCCAGATTATGCCTCCCTGCGGTCACTTGTCGCTAGTA<br>GCGGAACACTGGAGTTTAACAACGAGAGTTTCAATTGGGCTGGAGTGACCCAGAACGGCACCAGTTCTTCATGCAGACGAGGGTCAAATTCTTCATTTTTCTCTAGGCTCAACTGGCTCACTCATCTCAA<br>TAGTAAATACCCAGCTCTTAACGTGACTATGCCTAACAATGAGCAATTCGACAAGTTGTACATCTGGGGTGTGCACCATCCTGTCACAGATAAAGATCAGATCTTTCTTTACGCCCAGTCCTCCGGCCGTA<br>TAACAGTGTCAACTAAGCGCAGTCAGCAGGCTGTGATCCCTAACATCGGATACAGACCTAGGATACGCGACATCCCAAGCCGCATCTCCATCTACTGGACTATTGTAAAACCTGGCGACATCCTTCTGAT<br>CAACAGCACCGGCAACCTCATCGCTCCTCGCGGGTATTTTAAGATCCGCTCCGGCAAAAGTAGCATTATGAGGTCCGACGCACCTATCGGCAAGTGCAAGTCAGAGTGTATTACACCAAACGGAAGCAT<br>TCCCAATGACAAACCATTCCAGAACGTGAACCGCATCACCTATGGCGCCTGTCCAAGATACGTGAAGCAGTCTACCCTGAAACTGGCCACAGGTATGCGGAACGTGCCCGAGAGGCAAACCCGAGGCAT<br>CTTCGGGGCAATCGCAGGCTTCATCGAGAACGGTTGGGAAGGCATGGTCGATGGCTGGTACGGATTTAGACATCAGAACAGTGAAGGGAGAGGCCAAGCCGCTGATTTGAAGTCCACCCAGGCAGCCA<br>TCGACCAGATTAACGGCAAGCTGAACAGGTTGATAGGGAAAACAAATGAAAAGTTTCATCAAATTGAGAAGGAGTTTTCTGAGGTTGAGGGCAGGATTCAGGATCTGGAAAAGTATGTGGAAGATACT<br>AAGATCGATCTGTGGAGCTATAACGCCGAGCTGCTGGTAGCCCTGGAGAATCAGCACACAATTGACCTCACAGATTCTGAGATGAACAAACTGTTCGAAAAGACTAAGAAGCAACTCAGGGAAAATGC<br>AGAAGATATGGGCAATGGCTGTTTCAAAATCTATCATAAGTGTGACAATGCTTGTATCGGTTCCATCAGGAACGGCACTTATGACCATGACGTCTACAGGGATGAAGCCCTGAATAACCGGTTTCAGATC<br>AAGGGAGTCGAACTGAAGAGCGGATACAAAGATGGCTCTGGT**GCAATTGGTGGCTACATTCCAGAGGCCCCCAGAGATGGACAAGCATACGTGAGAAAGGATGGCGAATGGGTACTCTTGAGC**<br>**ACTTTTCTCTAA**
	Amino acid	MKTIIALSYILCLVFAQKLPGNDNSTATLCLGHHAVPNGTIVKTITNDRIEVTNATELVQNSSIGEICDSPHQILDGENCTLIDALLGDPQCDGFQNKKWDLFVERSKAYSNCYPYDVPDYASLRSLVASSGTLEFNN<br>ESFNWAGVTQNGTSSSCRRGSNSSFFSRLNWLTHLNSKYPALNVTMPNNEQFDKLYIWGVHHPVTDKDQIFLYAQSSGRITVSTKRSQQAVIPNIGYRPRIRDIPSRISIYWTIVKPGDILLINSTGNLIAPRGYFKIRS<br>GKSSIMRSDAPIGKCKSECITPNGSIPNDKPFQNVNRITYGACPRYVKQSTLKLATGMRNVPERQTRGIFGAIAGFIENGWEGMVDGWYGFRHQNSEGRGQAADLKSTQAAIDQINGKLNRLIGKTNEKFHQIEKE<br>FSEVEGRIQDLEKYVEDTKIDLWSYNAELLVALENQHTIDLTDSEMNKLFEKTKKQLRENAEDMGNGCFKIYHKCDNACIGSIRNGTYDHDVYRDEALNNRFQIKGVELKSGYKD**GSGAIGGYIPEAPRDGQA**<br>**YVRKDGEWVLLSTFL***
A/Switzerland/9715293/2013(H3N2) - H3 6HB	DNA	ATGAAGACCATCATCGCTCTGTCATACATCCTTTGTCTGGTGTTTGCCCAGAAGCTGCCAGGTAATGATAACTCAACAGCTACTCTTTGTCTGGGTCATCATGCAGTGCCCAACGGCACCATTGTCAAGAC<br>CATCACCAACGATCGGATAGAAGTGACCAACGCCACCGAATTGGTGCAGAACAGCTCAATCGGAGAGATCTGCGATAGTCCTCATCAAATTTTGGACGGAGAAAACTGCACCTTGATAGATGCTTTGCT<br>GGGCGATCCTCAGTGCGACGGCTTCCAAAACAAGAAGTGGGACCTTTTTGTGGAGCGCTCTAAGGCCTATTCCAATTGCTATCCATACGATGTTCCAGATTATGCCTCCCTGCGGTCACTTGTCGCTAGTA<br>GCGGAACACTGGAGTTTAACAACGAGAGTTTCAATTGGGCTGGAGTGACCCAGAACGGCACCAGTTCTTCATGCAGACGAGGGTCAAATTCTTCATTTTTCTCTAGGCTCAACTGGCTCACTCATCTCAA<br>TAGTAAATACCCAGCTCTTAACGTGACTATGCCTAACAATGAGCAATTCGACAAGTTGTACATCTGGGGTGTGCACCATCCTGTCACAGATAAAGATCAGATCTTTCTTTACGCCCAGTCCTCCGGCCGTA<br>TAACAGTGTCAACTAAGCGCAGTCAGCAGGCTGTGATCCCTAACATCGGATACAGACCTAGGATACGCGACATCCCAAGCCGCATCTCCATCTACTGGACTATTGTAAAACCTGGCGACATCCTTCTGAT<br>CAACAGCACCGGCAACCTCATCGCTCCTCGCGGGTATTTTAAGATCCGCTCCGGCAAAAGTAGCATTATGAGGTCCGACGCACCTATCGGCAAGTGCAAGTCAGAGTGTATTACACCAAACGGAAGCAT<br>TCCCAATGACAAACCATTCCAGAACGTGAACCGCATCACCTATGGCGCCTGTCCAAGATACGTGAAGCAGTCTACCCTGAAACTGGCCACAGGTATGCGGAACGTGCCCGAGAGGCAAACCCGAGGCAT<br>CTTCGGGGCAATCGCAGGCTTCATCGAGAACGGTTGGGAAGGCATGGTCGATGGCTGGTACGGATTTAGACATCAGAACAGTGAAGGGAGAGGCCAAGCCGCTGATTTGAAGTCCACCCAGGCAGCCA<br>TCGACCAGATTAACGGCAAGCTGAACAGGTTGATAGGGAAAACAAATGAAAAGTTTCATCAAATTGAGAAGGAGTTTTCTGAGGTTGAGGGCAGGATTCAGGATCTGGAAAAGTATGTGGAAGATACT<br>AAGATCGATCTGTGGAGCTATAACGCCGAGCTGCTGGTAGCCCTGGAGAATCAGCACACAATTGACCTCACAGATTCTGAGATGAACAAACTGTTCGAAAAGACTAAGAAGCAACTCAGGGAAAATGC<br>AGAAGATATGGGCAATGGCTGTTTCAAAATCTATCATAAGTGTGACAATGCTTGTATCGGTTCCATCAGGAACGGCACTTATGACCATGACGTCTACAGGGATGAAGCCCTGAATAACCGGTTTCAGATC<br>AAGGGAGTCGAACTGAAGAGCGGATACAAAGATGGCTCTGGT**ATAGTGCAGCAGCAGAACAATTTGCTGAGGGCTATTGAGGCGCAACAGCATCTGTTGCAACTCACAGTCTGGGGCATCAAGC**<br>**AGCTCCAAGCAAGAATCCTAGCTGGTGGCTCCGGCGGTCACACGACCTGGATGGAGTGGGACAGAGAAATTAACAATTACACAAGCTTAATACACTCCTTAATTGAAGAATCGCAAAACCAG**<br>**CAAGAAAAGAATGAACAAGAATTATTGGAATAG**

	Amino acid	MKTIIALSYILCLVFAQKLPGNDNSTATLCLGHHAVPNGTIVKTITNDRIEVTNATELVQNSSIGEICDSPHQILDGENCTLIDALLGDPQCDGFQNKKWDLFVERSKAYSNCYPYDVPDYASLRSLVASSGTLEFNN<br>ESFNWAGVTQNGTSSSCRRGSNSSFFSRLNWLTHLNSKYPALNVTMPNNEQFDKLYIWGVHHPVTDKDQIFLYAQSSGRITVSTKRSQQAVIPNIGYRPRIRDIPSRISIYWTIVKPGDILLINSTGNLIAPRGYFKIRS<br>GKSSIMRSDAPIGKCKSECITPNGSIPNDKPFQNVNRITYGACPRYVKQSTLKLATGMRNVPERQTRGIFGAIAGFIENGWEGMVDGWYGFRHQNSEGRGQAADLKSTQAAIDQINGKLNRLIGKTNEKFHQIEKE<br>FSEVEGRIQDLEKYVEDTKIDLWSYNAELLVALENQHTIDLTDSEMNKLFEKTKKQLRENAEDMGNGCFKIYHKCDNACIGSIRNGTYDHDVYRDEALNNRFQIKGVELKSGYKD**GSGIVQQQNNLLRAIEAQ**<br>**QHLLQLTVWGIKQLQARILAGGSGGHTTWMEWDREINNYTSLIHSLIEESQNQQEKNEQELLE*****
A/California/04/2009(H1N1pdm) - H1 Sol	DNA	ATGAAAGTAAAACTACTGGTCCTGTTATGCACATTCGCAACAGCTAACGCCGACACACTTTGTATCGGTTACCATGCCAATAATTCCACTGACACCGTCGACACAGTGCTGGAAAAGAACGTCACAGTCA<br>CACACTCAGTCAATCTGCTGGAAGACAAGCACAACGGCAAGCTGTGCAAGCTGCGCGGAGTGGCACCTTTGCATCTGGGAAAGTGCAATATCGCTGGCTGGATTCTGGGCAATCCTGAGTGCGAATCCC<br>TGAGCACCGCCTCTAGCTGGTCTTACATTGTCGAAACTCCTTCAAGTGACAACGGGACTTGCTACCCTGGGGACTTCATCGATTACGAAGAATTGCGTGAGCAGCTGAGCAGCGTGAGTTCATTTGAACG<br>CTTTGAGATTTTCCCTAAGACCTCCTCCTGGCCCAATCACGACTCCAACAAGGGAGTCACTGCCGCATGTCCCCATGCTGGAGCAAAATCTTTTTACAAAAACCTCATCTGGCTTGTCAAGAAAGGAAAC<br>TCATATCCAAAGCTCAGTAAGTCCTATATTAACGACAAAGGAAAGGAGGTTCTCGTTCTGTGGGGCATTCACCATCCTTCCACATCCGCAGATCAGCAGTCCCTCTATCAAAACGCCGATACCTATGTGTT<br>TGTCGGGTCATCCCGGTATTCAAAAAAATTTAAGCCTGAAATCGCCATTAGGCCAAAGGTGCGAGATCAGGAAGGACGCATGAATTACTATTGGACTCTTGTGGAGCCTGGCGATAAGATCACATTCGA<br>GGCAACTGGCAACCTGGTGGTTCCCAGGTACGCTTTTGCCATGGAGCGTAACGCTGGAAGTGGTATCATTATCTCTGATACCCCTGTGCATGATTGTAACACTACCTGTCAAACCCCTAAGGGAGCTATT<br>AACACCTCACTCCCCTTTCAAAATATTCATCCAATTACTATTGGCAAATGTCCTAAGTATGTTAAATCCACCAAGCTCCGTCTCGCCACCGGACTGCGCAACATTCCATCCATACAGTCTCGTGGATTGTTT<br>GGGGCTATAGCTGGCTTTATAGAGGGCGGATGGACAGGTATGGTTGACGGGTGGTACGGCTACCACCACCAGAACGAACAGGGAAGCGGGTATGCCGCCGACTTGAAATCAACACAGAACGCTATCGA<br>CGAGATAACCAACAAAGTGAACTCAGTCATAGAAAAGATGAACACACAGTTCACAGCAGTGGGTAAGGAGTTTAACCATCTCGAGAAAAGGATTGAGAATCTGAACAAAAAGGTCGACGATGGATTCC<br>TGGATATTTGGACTTACAATGCAGAGCTGCTGGTTCTTCTCGAGAATGAGCGCACTCTGGATTATCACGACTCTAACGTGAAAAACCTCTACGAGAAGGTCCGTTCACAGCTGAAGAATAATGCAAAAGA<br>GATCGGGAACGGGTGTTTTGAATTTTATCACAAATGCGACAACACCTGCATGGAGTCTGTGAAGAATGGGACTTATGACTACCCTAAGTATTCCGAGGAAGCTAAGCTCAACCGAGAGGAGATCGACGG<br>GGTCAAGCTTGAAAGCACCCGG
	Amino acid	MKVKLLVLLCTFATANADTLCIGYHANNSTDTVDTVLEKNVTVTHSVNLLEDKHNGKLCKLRGVAPLHLGKCNIAGWILGNPECESLSTASSWSYIVETPSSDNGTCYPGDFIDYEELREQLSSVSSFERFEIFPKT<br>SSWPNHDSNKGVTAACPHAGAKSFYKNLIWLVKKGNSYPKLSKSYINDKGKEVLVLWGIHHPSTSADQQSLYQNADTYVFVGSSRYSKKFKPEIAIRPKVRDQEGRMNYYWTLVEPGDKITFEATGNLVVPRY<br>AFAMERNAGSGIIISDTPVHDCNTTCQTPKGAINTSLPFQNIHPITIGKCPKYVKSTKLRLATGLRNIPSIQSRGLFGAIAGFIEGGWTGMVDGWYGYHHQNEQGSGYAADLKSTQNAIDEITNKVNSVIEKMNTQFT<br>AVGKEFNHLEKRIENLNKKVDDGFLDIWTYNAELLVLLENERTLDYHDSNVKNLYEKVRSQLKNNAKEIGNGCFEFYHKCDNTCMESVKNGTYDYPKYSEEAKLNREEIDGVKLESTR*
A/California/04/2009(H1N1pdm) - H1 Foldon (Igκ signal sequence)	DNA	*ATGGGATGGAGCTGTATCATCCTCTTCTTGGTAGCAACAGCTACAGGTGTCCACTCCGAG*GACACACTTTGTATCGGTTACCATGCCAATAATTCCACTGACACCGTCGACACAGTGCTGGAAAAGAACGTCA<br>CAGTCACACACTCAGTCAATCTGCTGGAAGACAAGCACAACGGCAAGCTGTGCAAGCTGCGCGGAGTGGCACCTTTGCATCTGGGAAAGTGCAATATCGCTGGCTGGATTCTGGGCAATCCTGAGTGCG<br>AATCCCTGAGCACCGCCTCTAGCTGGTCTTACATTGTCGAAACTCCTTCAAGTGACAACGGGACTTGCTACCCTGGGGACTTCATCGATTACGAAGAATTGCGTGAGCAGCTGAGCAGCGTGAGTTCATT<br>TGAACGCTTTGAGATTTTCCCTAAGACCTCCTCCTGGCCCAATCACGACTCCAACAAGGGAGTCACTGCCGCATGTCCCCATGCTGGAGCAAAATCTTTTTA<br>CAAAAACCTCATCTGGCTTGTCAAGAAAGGAAACTCATATCCAAAGCTCAGTAAGTCCTATATTAACGACAAAGGAAAGGAGGTTCTCGTTCTGTGGGGCATTCACCATCCTTCCACATCCGCAGATCAG<br>CAGTCCCTCTATCAAAACGCCGATACCTATGTGTTTGTCGGGTCATCCCGGTATTCAAAAAAATTTAAGCCTGAAATCGCCATTAGGCCAAAGGTGCGAGATCAGGAAGGACGCATGAATTACTATTGGA<br>CTCTTGTGGAGCCTGGCGATAAGATCACATTCGAGGCAACTGGCAACCTGGTGGTTCCCAGGTACGCTTTTGCCATGGAGCGTAACGCTGGAAGTGGTATCATTATCTCTGATACCCCTGTGCATGATTGT<br>AACACTACCTGTCAAACCCCTAAGGGAGCTATTAACACCTCACTCCCCTTTCAAAATATTCATCCAATTACTATTGGCAAATGTCCTAAGTATGTTAAATCCACCAAGCTCCGTCTCGCCACCGGACTGCG<br>CAACATTCCATCCATACAGTCTCGTGGATTGTTTGGGGCTATAGCTGGCTTTATAGAGGGCGGATGGACAGGTATGGTTGACGGGTGGTACGGCTACCACCACCAGAACGAACAGGGAAGCGGGTATGC<br>CGCCGACTTGAAATCAACACAGAACGCTATCGACGAGATAACCAACAAAGTGAACTCAGTCATAGAAAAGATGAACACACAGTTCACAGCAGTGGGTAAGGAGTTTAACCATCTCGAGAAAAGGATTG<br>AGAATCTGAACAAAAAGGTCGACGATGGATTCCTGGATATTTGGACTTACAATGCAGAGCTGCTGGTTCTTCTCGAGAATGAGCGCACTCTGGATTATCACGACTCTAACGTGAAAAACCTCTACGAGAA<br>GGTCCGTTCACAGCTGAAGAATAATGCAAAAGAGATCGGGAACGGGTGTTTTGAATTTTATCACAAATGCGACAACACCTGCATGGAGTCTGTGAAGAATGGGACTTATGACTACCCTAAGTATTCCGA<br>GGAAGCTAAGCTCAACCGAGAGGAGATCGACGGGGTCAAGCTTGAAAGCACCCGGGGGTCA**GCAATTGGTGGCTACATTCCAGAGGCCCCCAGAGATGGACAAGCATACGTGAGAAAGGATGGC**<br>**GAATGGGTACTCTTGAGCACTTTTCTGTAA**
	Amino acid	*MGWSCIILFLVATATGVHSE*DTLCIGYHANNSTDTVDTVLEKNVTVTHSVNLLEDKHNGKLCKLRGVAPLHLGKCNIAGWILGNPECESLSTASSWSYIVETPSSDNGTCYPGDFIDYEELREQLSSVSSFERFEIFPK<br>TSSWPNHDSNKGVTAACPHAGAKSFYKNLIWLVKKGNSYPKLSKSYINDKGKEVLVLWGIHHPSTSADQQSLYQNADTYVFVGSSRYSKKFKPEIAIRPKVRDQEGRMNYYWTLVEPGDKITFEATGNLVVPRY<br>AFAMERNAGSGIIISDTPVHDCNTTCQTPKGAINTSLPFQNIHPITIGKCPKYVKSTKLRLATGLRNIPSIQSRGLFGAIAGFIEGGWTGMVDGWYGYHHQNEQGSGYAADLKSTQNAIDEITNKVNSVIEKMNTQFT<br>AVGKEFNHLEKRIENLNKKVDDGFLDIWTYNAELLVLLENERTLDYHDSNVKNLYEKVRSQLKNNAKEIGNGCFEFYHKCDNTCMESVKNGTYDYPKYSEEAKLNREEIDGVKLESTRGGSAI**GGYIPEAPRD**<br>**GQAYVRKDGEWVLLSTFL***
A/California/04/2009(H1N1pdm) - H1 6HB (Igκ signal sequence)	DNA	*ATGGGATGGAGCTGTATCATCCTCTTCTTGGTAGCAACAGCTACAGGTGTCCACTCCGAG*GACACACTTTGTATCGGTTACCATGCCAATAATTCCACTGACACCGTCGACACAGTGCTGGAAAAGAACGTCA<br>CAGTCACACACTCAGTCAATCTGCTGGAAGACAAGCACAACGGCAAGCTGTGCAAGCTGCGCGGAGTGGCACCTTTGCATCTGGGAAAGTGCAATATCGCTGGCTGGATTCTGGGCAATCCTGAGTGCG<br>AATCCCTGAGCACCGCCTCTAGCTGGTCTTACATTGTCGAAACTCCTTCAAGTGACAACGGGACTTGCTACCCTGGGGACTTCATCGATTACGAAGAATTGCGTGAGCAGCTGAGCAGCGTGAGTTCATT<br>TGAACGCTTTGAGATTTTCCCTAAGACCTCCTCCTGGCCCAATCACGACTCCAACAAGGGAGTCACTGCCGCATGTCCCCATGCTGGAGCAAAATCTTTTTACAAAAACCTCATCTGGCTTGTCAAGAAA<br>GGAAACTCATATCCAAAGCTCAGTAAGTCCTATATTAACGACAAAGGAAAGGAGGTTCTCGTTCTGTGGGGCATTCACCATCCTTCCACATCCGCAGATCAGCAGTCCCTCTATCAAAACGCCGATACCT<br>ATGTGTTTGTCGGGTCATCCCGGTATTCAAAAAAATTTAAGCCTGAAATCGCCATTAGGCCAAAGGTGCGAGATCAGGAAGGACGCATGAATTACTATTGGACTCTTGTGGAGCCTGGCGATAAGATCAC<br>ATTCGAGGCAACTGGCAACCTGGTGGTTCCCAGGTACGCTTTTGCCATGGAGCGTAACGCTGGAAGTGGTATCATTATCTCTGATACCCCTGTGCATGATTGTAACACTACCTGTCAAACCCCTAAGGGA<br>GCTATTAACACCTCACTCCCCTTTCAAAATATTCATCCAATTACTATTGGCAAATGTCCTAAGTATGTTAAATCCACCAAGCTCCGTCTCGCCACCGGACTGCGCAACATTCCATCCATACAGTCTCGTGG<br>ATTGTTTGGGGCTATAGCTGGCTTTATAGAGGGCGGATGGACAGGTATGGTTGACGGGTGGTACGGCTACCACCACCAGAACGAACAGGGAAGCGGGTATGCCGCCGACTTGAAATCAACACAGAACG<br>CTATCGACGAGATAACCAACAAAGTGAACTCAGTCATAGAAAAGATGAACACACAGTTCACAGCAGTGGGTAAGGAGTTTAACCATCTCGAGAAAAGGATTGAGAATCTGAACAAAAAGGTCGACGAT<br>GGATTCCTGGATATTTGGACTTACAATGCAGAGCTGCTGGTTCTTCTCGAGAATGAGCGCACTCTGGATTATCACGACTCTAACGTGAAAAACCTCTACGAGAAGGTCCGTTCACAGCTGAAGAATAATG<br>CAAAAGAGATCGGGAACGGGTGTTTTGAATTTTATCACAAATGCGACAACACCTGCATGGAGTCTGTGAAGAATGGGACTTATGACTACCCTAAGTATTCCGAGGAAGCTAAGCTCAACCGAGAGGAGA<br>TCGACGGGGTCAAGCTTGAAAGCACCCGG**GGCGGGTCTGGAATCGTGCAGCAGCAGAACAACCTGCTGAGAGCCATCGAGGCCCAGCAGCATCTGCTGCAGTTGACCGTGTGGGGCATCAAG**<br>**CAGCTGCAGGCCAGAATTCTCGCTGGCGGATCTGGCGGCCATACCACCTGGATGGAATGGGACAGAGAGATCAACAACTACACCAGCCTGATCCACAGCCTGATTGAGGAATCCCAGAACCA**<br>**GCAAGAGAAGAATGAGCAAGAGCTGCTCGAGTAA**
	Amino acid	*MGWSCIILFLVATATGVHSE*DTLCIGYHANNSTDTVDTVLEKNVTVTHSVNLLEDKHNGKLCKLRGVAPLHLGKCNIAGWILGNPECESLSTASSWSYIVETPSSDNGTCYPGDFIDYEELREQLSSVSSFERFEIFPK<br>TSSWPNHDSNKGVTAACPHAGAKSFYKNLIWLVKKGNSYPKLSKSYINDKGKEVLVLWGIHHPSTSADQQSLYQNADTYVFVGSSRYSKKFKPEIAIRPKVRDQEGRMNYYWTLVEPGDKITFEATGNLVVPRY<br>AFAMERNAGSGIIISDTPVHDCNTTCQTPKGAINTSLPFQNIHPITIGKCPKYVKSTKLRLATGLRNIPSIQSRGLFGAIAGFIEGGWTGMVDGWYGYHHQNEQGSGYAADLKSTQNAIDEITNKVNSVIEKMNTQFT

		AVGKEFNHLEKRIENLNKKVDDGFLDIWTYNAELLVLLENERTLDYHDSNVKNLYEKVRSQLKNNAKEIGNGCFEFYHKCDNTCMESVKNGTYDYPKYSEEAKLNREEIDGVKLESTR**GGSGIVQQQNNLLR**<br>**AIEAQQHLLQLTVWGIKQLQARILAGGSGGTTWMEWDREINNYTSLIHSLIEESQNQQEKNEQELLE***
A/Vietnam/1202/2004(H5N1) - H5 Sol	DNA	ATGGAACGGATCGTGCTGCTGTTCGCCATCGTGTCCCTCGTGAAGTCCGACCAGATCTGCATCGGCTACCACGCCAACAACTCCACCGAACAGGTGGACACCATCATGGAAAAGAACGTGACCGTGACC<br>CACGCCCAGGACATTCTGGAAAAGACCCACAACGGCAAGCTGTGCGACCTGGACGGCGTGAAGCCCCTGATCCTGAGAGACTGTTCTGTGGCCGGCTGGCTGCTGGGCAACCCTATGTGCGACGAGTTC<br>ATCAACGTGCCCGAGTGGTCCTACATCGTGGAAAAGGCCAACCCCGTGAACGACCTGTGCTACCCCGGCGACTTCAACGACTACGAGGAACTGAAACATCTGCTGTCCCGGATCAACCACTTCGAGAAG<br>ATCCAGATTATCCCCAAGTCCTCCTGGTCCTCCCACGAGGCTTCTCTGGGCGTGTCCTCCGCATGTCCATACCAGGGCAAGTCCAGCTTCTTCCGGAACGTCGTGTGGCTGATCAAGAAGAACTCCACCTA<br>CCCCACCATCAAGCGGTCCTACAACAACACCAACCAGGAAGATCTGCTGGTGCTGTGGGGCATCCACCACCCTAATGATGCCGCCGAGCAGACCAAGCTGTACCAGAACCCCACCACCTACATCTCCGT<br>GGGCACCTCCACCCTGAACCAGCGGCTGGTGCCTAGAATCGCCACCCGGTCCAAAGTGAACGGCCAGTCCGGCAGAATGGAATTTTTCTGGACCATCCTGCGGCCCAACGACGCCATCAACTTCGAGTC<br>CAACGGCAACTTTATCGCCCCCGAGTACGCCTACAAGATCGTGAAGAAGGGCGACTCTACCATCATGAAGTCTGAGCTGGAATACGGCAACTGCAACGCCAAGTGCCAGACCCCCATGGGCGCCATCAA<br>TTCCTCCATGCCCTTCCACAACATCCACCCCCTGACCATCGGCGAGTGCCCCAAATACGTGAAGTCTAACAGACTGGTGCTGGCCACCGGCCTGCGGAACTCTCCCCAGCGCGAGCGGAGAAGAAAGAA<br>GCGGGGCCTGTTTGGCGCTATCGCCGGCTTTATTGAGGGCGGCTGGCAGGGCATGGTGGACGGGTGGTACGGCTACCATCACTCCAACGAGCAGGGCTCTGGCTACGCCGCCGACAAAGAGTCCACCCA<br>GAAAGCCATCGACGGCGTGACCAACAAAGTGAACTCCATCATCGACAAGATGAACACCCAGTTCGAGGCCGTGGGCAGAGAGTTCAACAACCTGGAACGGCGGATCGAGAACCTGAACAAGAAAATG<br>GAAGATGGCTTCCTGGACGTGTGGACCTACAACGCCGAGCTGCTGGTGCTGATGGAAAACGAGCGGACCCTGGACTTCCACGACTCCAACGTGAAGAACCTGTACGACAAAGTGCGGCTGCAGCTGCGG<br>GACAACGCCAAAGAACTGGGCAACGGCTGCTTCGAGTTCTACCACAAGTGCGACAACGAGTGCATGGAATCCGTGCGGAACGGCACCTACGACTACCCCCAGTACTCTGAGGAAGCCCGGCTGAAGCG<br>GGAAGAGATCAGCGGAGTGAAGCTGGAATCCATCGGCTAA
	Amino acid	MERIVLLFAIVSLVKSDQICIGYHANNSTEQVDTIMEKNVTVTHAQDILEKTHNGKLCDLDGVKPLILRDCSVAGWLLGNPMCDEFINVPEWSYIVEKANPVNDLCYPGDFNDYEELKHLLSRINHFEKIQIIPKSS<br>WSSHEASLGVSSACPYQGKSSFFRNVVWLIKKNSTYPTIKRSYNNTNQEDLLVLWGIHHPNDAAEQTKLYQNPTTYISVGTSTLNQRLVPRIATRSKVNGQSGRMEFFWTILRPNDAINFESNGNFIAPEYAYKIVK<br>KGDSTIMKSELEYGNCNAKCQTPMGAINSSMPFHNIHPLTIGECPKYVKSNRLVLATGLRNSPQRERRRKKRGLFGAIAGFIEGGWQGMVDGWYGYHHSNEQGSGYAADKESTQKAIDGVTNKVNSIIDKMNTQ<br>FEAVGREFNNLERRIENLNKKMEDGFLDVWTYNAELLVLMENERTLDFHDSNVKNLYDKVRLQLRDNAKELGNGCFEFYHKCDNECMESVRNGTYDYPQYSEEARLKREEISGVKLESIG*
A/Vietnam/1202/2004(H5N1) - H5 6HB	DNA	ATGGAACGGATCGTGCTGCTGTTCGCCATCGTGTCCCTCGTGAAGTCCGACCAGATCTGCATCGGCTACCACGCCAACAACTCCACCGAACAGGTGGACACCATCATGGAAAAGAACGTGACCGTGACC<br>CACGCCCAGGACATTCTGGAAAAGACCCACAACGGCAAGCTGTGCGACCTGGACGGCGTGAAGCCCCTGATCCTGAGAGACTGTTCTGTGGCCGGCTGGCTGCTGGGCAACCCTATGTGCGACGAGTTC<br>ATCAACGTGCCCGAGTGGTCCTACATCGTGGAAAAGGCCAACCCCGTGAACGACCTGTGCTACCCCGGCGACTTCAACGACTACGAGGAACTGAAACATCTGCTGTCCCGGATCAACCACTTCGAGAAG<br>ATCCAGATTATCCCCAAGTCCTCCTGGTCCTCCCACGAGGCTTCTCTGGGCGTGTCCTCCGCATGTCCATACCAGGGCAAGTCCAGCTTCTTCCGGAACGTCGTGTGGCTGATCAAGAAGAACTCCACCTA<br>CCCCACCATCAAGCGGTCCTACAACAACACCAACCAGGAAGATCTGCTGGTGCTGTGGGGCATCCACCACCCTAATGATGCCGCCGAGCAGACCAAGCTGTACCAGAACCCCACCACCTACATCTCCGT<br>GGGCACCTCCACCCTGAACCAGCGGCTGGTGCCTAGAATCGCCACCCGGTCCAAAGTGAACGGCCAGTCCGGCAGAATGGAATTTTTCTGGACCATCCTGCGGCCCAACGACGCCATCAACTTCGAGTC<br>CAACGGCAACTTTATCGCCCCCGAGTACGCCTACAAGATCGTGAAGAAGGGCGACTCTACCATCATGAAGTCTGAGCTGGAATACGGCAACTGCAACGCCAAGTGCCAGACCCCCATGGGCGCCATCAA<br>TTCCTCCATGCCCTTCCACAACATCCACCCCCTGACCATCGGCGAGTGCCCCAAATACGTGAAGTCTAACAGACTGGTGCTGGCCACCGGCCTGCGGAACTCTCCCCAGCGCGAGCGGAGAAGAAAGAA<br>GCGGGGCCTGTTTGGCGCTATCGCCGGCTTTATTGAGGGCGGCTGGCAGGGCATGGTGGACGGGTGGTACGGCTACCATCACTCCAACGAGCAGGGCTCTGGCTACGCCGCCGACAAAGAGTCCACCCA<br>GAAAGCCATCGACGGCGTGACCAACAAAGTGAACTCCATCATCGACAAGATGAACACCCAGTTCGAGGCCGTGGGCAGAGAGTTCAACAACCTGGAACGGCGGATCGAGAACCTGAACAAGAAAATG<br>GAAGATGGCTTCCTGGACGTGTGGACCTACAACGCCGAGCTGCTGGTGCTGATGGAAAACGAGCGGACCCTGGACTTCCACGACTCCAACGTGAAGAACCTGTACGACAAAGTGCGGCTGCAGCTGCGG<br>GACAACGCCAAAGAACTGGGCAACGGCTGCTTCGAGTTCTACCACAAGTGCGACAACGAGTGCATGGAATCCGTGCGGAACGGCACCTACGACTACCCCCAGTACTCTGAGGAAGCCCGGCTGAAGCG<br>GGAAGAGATCAGCGGAGTGAAGCTGGAATCCATCGGCGGTGGGTCTGGT**ATAGTGCAGCAGCAGAACAATTTGCTGAGGGCTATTGAGGCGCAACAGCATCTGTTGCAACTCACAGTCTGGGGC**<br>**ATCAAGCAGCTCCAAGCAAGAATCCTAGCTGGTGGCTCCGGCGGTCACACGACCTGGATGGAGTGGGACAGAGAAATTAACAATTACACAAGCTTAATACACTCCTTAATTGAAGAATCGCA**<br>**AAACCAGCAAGAAAAGAATGAACAAGAATTATTGGAATAG**
	Amino acid	MERIVLLFAIVSLVKSDQICIGYHANNSTEQVDTIMEKNVTVTHAQDILEKTHNGKLCDLDGVKPLILRDCSVAGWLLGNPMCDEFINVPEWSYIVEKANPVNDLCYPGDFNDYEELKHLLSRINHFEKIQIIPKSS<br>WSSHEASLGVSSACPYQGKSSFFRNVVWLIKKNSTYPTIKRSYNNTNQEDLLVLWGIHHPNDAAEQTKLYQNPTTYISVGTSTLNQRLVPRIATRSKVNGQSGRMEFFWTILRPNDAINFESNGNFIAPEYAYKIVK<br>KGDSTIMKSELEYGNCNAKCQTPMGAINSSMPFHNIHPLTIGECPKYVKSNRLVLATGLRNSPQRERRRKKRGLFGAIAGFIEGGWQGMVDGWYGYHHSNEQGSGYAADKESTQKAIDGVTNKVNSIIDKMNTQ<br>FEAVGREFNNLERRIENLNKKMEDGFLDVWTYNAELLVLMENERTLDFHDSNVKNLYDKVRLQLRDNAKELGNGCFEFYHKCDNECMESVRNGTYDYPQYSEEARLKREEISGVKLESI**GGSGIVQQQNNLL**<br>**RAIEAQQHLLQLTVWGIKQLQARILAGGSGGHTTWMEWDREINNYTSLIHSLIEESQNQQEKNEQELLE***
A/Shanghai01/2014(H7N9) - H7 6HB (Igκ signal sequence)	DNA	*ATGGGATGGAGCTGTATCATCCTCTTCTTGGTAGCAACAGCTACAGGTGTCCACTCCGAG*GACAAAATTTGCCTGGGACACCATGCAGTTAGTAATGGAACAAAGGTCAACACCCTGACTGAGCGAGGAGTCG<br>AGGTGGTGAACGCAACAGAAACAGTTGAGAGAACCAATATTCCTCGCATCTGCTCCAAAGGCAAGCGCACAGTAGATCTGGGACAGTGTGGACTTCTGGGCACTATCACAGGCCCTCCCCAGTGTGACC<br>AGTTCCTGGAGTTCAGTGCCGATCTGATTATCGAAAGGCGAGAAGGCAGCGATGTGTGTTATCCTGGAAAGTTCGTGAACGAAGAGGCCTTGAGACAGATACTCAGGGAATCTGGCGGGATCGACAAAG<br>AGGCCATGGGGTTTACCTATTCCGGGATCAGGACTAACGGGGCCACCTCAGCATGCCGGCGGTCCGGCAGCTCATTTTATGCTGAGATGAAATGGCTGTTGAGTAACACAGACAACGCTGCTTTTCCCCA<br>AATGACTAAGTCCTACAAAAACACCCGCAAATCTCCTGCCTTGATTGTTTGGGGCATTCATCACAGTGTTAGCACTGCTGAGCAGACCAAGCTCTACGGGTCCGGCAATAAGTTGGTGACAGTGGGCTCT<br>TCCAACTACCAGCAGAGCTTCGTGCCCTCTCCCGGAGCAAGGCCCCAAGTAAACGGGCTTAGCGGGCGAATTGACTTCCACTGGTTGATGCTGAATCCCAATGACACCGTCACATTTAGCTTCAATGGGG<br>CTTTCATAGCCCCAGACCGCGCATCCTTTTTGCGCGGCAAGTCTATGGGCATCCAGTCCGGTGTCCAGGTGGATGCAAACTGCGAGGGCGACTGTTACCACTCTGGCGGTACCATTATATCCAACTTGCCC<br>TTCCAGAACATCGATTCCCGCGCCGTCGGGAAATGCCCCAGATACGTGAAGCAGCGTTCTCTGCTTCTCGCCACTGGTATGAAGAATGTGCCAGAAATTCCTAAAGGCCGGGGTCTGTTCGGCGCCATCG<br>CAGGCTTCATTGAGAACGGATGGGAGGGACTGATCGACGGTTGGTACGGCTTCAGACACCAGAACGCACAGGGAGAAGGCACAGCAGCCGACTACAAAAGTACTCAGTCTGCCATCGACCAGATCACA<br>GGAAAATTGAACCGCCTCATCGAAAAAACAAATCAGCAATTTGAACTCATAGATAACGAGTTCAACGAGGTGGAAAAACAGATCGGTAACGTGATAAACTGGACACGAGACAGCATCACCGAGGTGTG<br>GTCTTATAATGCAGAGCTCCTCGTCGCAATGGAGAATCAGCATACCATCGATCTGGCAGATAGTGAAATGGATAAGTTGTACGAAAGGGTCAAGAGACAGCTCCGGGAAAATGCAGAGGAAGATGGAA<br>CCGGTTGTTTTGAAATATTCCACAAATGCGATGATGATTGCATGGCTAGCATCCGAAATAACACTTACGACCATTCCAAGTATCGGGAAGAAGCTATGCAGAACCGTATCCAGATCGACCCAGTGAAACT<br>TTCTAGCGGTTACAAAGACGTGATACTC**GGCGGGTCTGGAATCGTGCAGCAGCAGAACAACCTGCTGAGAGCCATCGAGGCCCAGCAGCATCTGCTGCAGTTGACCGTGTGGGGCATCAAGCA**<br>**GCTGCAGGCCAGAATTCTCGCTGGCGGATCTGGCGGCCATACCACCTGGATGGAATGGGACAGAGAGATCAACAACTACACCAGCCTGATCCACAGCCTGATTGAGGAATCCCAGAACCAG**<br>**CAAGAGAAGAATGAGCAAGAGCTGCTCGAGTAA**
	Amino acid	*MGWSCIILFLVATATGVHSE*DKICLGHHAVSNGTKVNTLTERGVEVVNATETVERTNIPRICSKGKRTVDLGQCGLLGTITGPPQCDQFLEFSADLIIERREGSDVCYPGKFVNEEALRQILRESGGIDKEAMGFTYSG<br>IRTNGATSACRRSGSSFYAEMKWLLSNTDNAAFPQMTKSYKNTRKSPALIVWGIHHSVSTAEQTKLYGSGNKLVTVGSSNYQQSFVPSPGARPQVNGLSGRIDFHWLMLNPNDTVTFSFNGAFIAPDRASFLRGK<br>SMGIQSGVQVDANCEGDCYHSGGTIISNLPFQNIDSRAVGKCPRYVKQRSLLLATGMKNVPEIPKGRGLFGAIAGFIENGWEGLIDGWYGFRHQNAQGEGTAADYKSTQSAIDQITGKLNRLIEKTNQQFELIDNE<br>FNEVEKQIGNVINWTRDSITEVWSYNAELLVAMENQHTIDLADSEMDKLYERVKRQLRENAEEDGTGCFEIFHKCDDDCMASIRNNTYDHSKYREEAMQNRIQIDPVKLSSGYKDVIL**GGSGIVQQQNNLLRAI**<br>**EAQQHLLQLTVWGIKQLQARILAGGSGGHTTWMEWDREINNYTSLIHSLIEESQNQQEKNEQELLE***

A/Chicken/Quingdao/020/2014(H9N2) - H9 6HB (Ig*κ* signal sequence)	DNA	*ATGGGATGGAGCTGTATCATCCTCTTCTTGGTAGCAACAGCTACAGGTGTCCACTCCGAG*GACAAAATCTGCATTGGATATCAGAGTACCAACTCCACCGAGACCGTGGATACCTTGACCGAAAACAACGTGC<br>CTGTCACCCACGCTAAAGAGCTGCTGCACACTGAGCACAATGGCATGCTGTGTGCCACTAGCCTGGGCCAGCCACTCGTCCTGGATACCTGCACCATAGAAGGCCTTATTTACGGTAATCCATCCTGCGA<br>TCTCAGCCTGGAAGGTCGGGAATGGAGCTATATTGTCGAACGGCCATCTGCCGTGAACGGTCTGTGCTATCCCGGGAACGTTGAAAACCTGGAAGAGCTTCGTAGCTTGTTTTCCAGTGCTAGGTCTTAT<br>CAGAGAATCCAGATTTTTCCAGACACCATCTGGAACGTCAGCTATGATGGGACCAGTAATGCCTGTAGTGGCTCTTTTTATCGCTCTATCAGGTGGCTTACACGCAAAAATGGAGATTACCCAATTCAAG<br>ACGCCCAGTACACTAACAATCAAGGGAAGAATATACTGTTTATGTGGGGCATCAACCAACCTACCAATCAGACCACTCAGCGTGACCTCTACACAAGAACTGACACCACCACTAGCGTGGCAACCGAAG<br>AGATCAACAGAATTTTCAAGCCTCTGATCGGCCCACGGCCTCTGGTCAACGGGCTGATGGGGCGAATTGACTACTATTGGTCAGTACTCAAGCCCGGCCAGACTTTGAGGATTAAATCTGATGGTAATTT<br>GATCGCCCCTTGGTATGGACACATCTTGTCTGGCGAGTCCCACGGTCGGATTCTGAAAACTGATTTGAAGAGGGGATCTTGCACCGTGCAGTGCCAAACAGAAAAAGGCGGCCTCAACACCACACTTCC<br>ATTCCAAAACGTCAGTAAGTATGCTTTTGGAAACTGCTCCAAGTATATTGGGATCAAGTCACTGAAGTTGGCAGTCGGACTGCGCAACGTCCCTAGCCGGTCCAGTAGGGGACTGTTCGGTGCTATCGCC<br>GGTTTCATCGAGGGCGGCTGGTCCGGTCTGGTGGCCGGATGGTACGGCTTTCAGCATAGTAATGATCAGGGGGTCGGTATGGCAGCCGACAGAGACTCCACCCAGAAGGCTATCGATAAAATCACAAGC<br>AAGGTGAACAATATTGTAGACAAAATGAACAAGCAGTACGAGATTATCGATCACGAGTTCAGCGAGGTCGAGACCCGTCTGAACATGATTAATAACAAAATAGACGACCAGATCCAGGACATTTGGGC<br>ATACAACGCAGAGCTTCTGGTGCTCCTGGAGAATCAGAAGACCCTCGATGAGCACGACGCCAACGTTAATAACCTGTATAATAAGGTGAAACGGGCCTTGGGAAGCAACGCCGTGGAGGACGGCAAGG<br>GCTGCTTTGAGCTTTACCATAAATGCGATGACCAGTGCATGGAAACAATCAGGAACGGGACATACAACCGTAGAAAGTACCAAGAGGAGTCTAAACTTGAGAGGCAGAAAATAGAGGGCGTTAAACTG<br>GAGAGCGAAGGAACTTACAAGATACTCGGCGGGTCTGGT**ATAGTGCAGCAGCAGAACAATTTGCTGAGGGCTATTGAGGCGCAACAGCATCTGTTGCAACTCACAGTCTGGGGCATCAAGCAGC**<br>**TCCAAGCAAGAATCCTAGCTGGTGGCTCCGGCGGTCACACGACCTGGATGGAGTGGGACAGAGAAATTAACAATTACACAAGCTTAATACACTCCTTAATTGAAGAATCGCAAAACCAGCAA**<br>**GAAAAGAATGAACAAGAATTATTGGAATAG**
	Amino acid	*MGWSCIILFLVATATGVHSE*DKICIGYQSTNSTETVDTLTENNVPVTHAKELLHTEHNGMLCATSLGQPLVLDTCTIEGLIYGNPSCDLSLEGREWSYIVERPSAVNGLCYPGNVENLEELRSLFSSARSYQRIQIFPDT<br>IWNVSYDGTSNACSGSFYRSIRWLTRKNGDYPIQDAQYTNNQGKNILFMWGINQPTNQTTQRDLYTRTDTTTSVATEEINRIFKPLIGPRPLVNGLMGRIDYYWSVLKPGQTLRIKSDGNLIAPWYGHILSGESHG<br>RILKTDLKRGSCTVQCQTEKGGLNTTLPFQNVSKYAFGNCSKYIGIKSLKLAVGLRNVPSRSSRGLFGAIAGFIEGGWSGLVAGWYGFQHSNDQGVGMAADRDSTQKAIDKITSKVNNIVDKMNKQYEIIDHEFS<br>EVETRLNMINNKIDDQIQDIWAYNAELLVLLENQKTLDEHDANVNNLYNKVKRALGSNAVEDGKGCFELYHKCDDQCMETIRNGTYNRRKYQEESKLERQKIEGVKLESEGTYKIL**GGSGIVQQQNNLLRAIE**<br>**AQQHLLQLTVWGIKQLQARILAGGSGGHTTWMEWDREINNYTSLIHSLIEESQNQQEKNEQELLE***
