# Supplementary material for: Development of molecular clamp stabilized hemagglutinin vaccines for Influenza A viruses
Source: NPJ Vaccines. 2021 Nov 8;6:135. doi: 10.1038/s41541-021-00395-4 (PMC8575991; doi:10.1038/s41541-021-00395-4)
Supplement: Supplementary file 2 — Reporting Summary [file 41541_2021_395_MOESM2_ESM.pdf]

nature portfolio

Corresponding author(s): Professor Keith Chappell

Last updated by author(s): 13.07.2021

Reporting Summary

Nature Portfolio wishes to improve the reproducibility of the work that we publish. This form provides structure for consistency and transparency in reporting. For further information on Nature Portfolio policies, see our Editorial Policies and the Editorial Policy Checklist.

Statistics

For all statistical analyses, confirm that the following items are present in the figure legend, table legend, main text, or Methods section.

n/a	Confirmed	
☐	☒	The exact sample size (*n*) for each experimental group/condition, given as a discrete number and unit of measurement
☒	☐	A statement on whether measurements were taken from distinct samples or whether the same sample was measured repeatedly
☐	☒	The statistical test(s) used AND whether they are one- or two-sided<br>*Only common tests should be described solely by name; describe more complex techniques in the Methods section.*
☒	☐	A description of all covariates tested
☐	☒	A description of any assumptions or corrections, such as tests of normality and adjustment for multiple comparisons
☐	☒	A full description of the statistical parameters including central tendency (e.g. means) or other basic estimates (e.g. regression coefficient) AND variation (e.g. standard deviation) or associated estimates of uncertainty (e.g. confidence intervals)
☒	☐	For null hypothesis testing, the test statistic (e.g. *F*, *t*, *r*) with confidence intervals, effect sizes, degrees of freedom and *P* value noted<br>*Give P values as exact values whenever suitable.*
☒	☐	For Bayesian analysis, information on the choice of priors and Markov chain Monte Carlo settings
☒	☐	For hierarchical and complex designs, identification of the appropriate level for tests and full reporting of outcomes
☒	☐	Estimates of effect sizes (e.g. Cohen's *d*, Pearson's *r*), indicating how they were calculated

Our web collection on statistics for biologists contains articles on many of the points above.

Software and code

Policy information about availability of computer code

Data collection	*Provide a description of all commercial, open source and custom code used to collect the data in this study, specifying the version used OR state that no software was used.*
Data analysis	Data analysis was performed using Graphpad Prism version 9.12 (225).

For manuscripts utilizing custom algorithms or software that are central to the research but not yet described in published literature, software must be made available to editors and reviewers. We strongly encourage code deposition in a community repository (e.g. GitHub). See the Nature Portfolio guidelines for submitting code & software for further information.

Data

Policy information about availability of data

All manuscripts must include a data availability statement. This statement should provide the following information, where applicable:

- Accession codes, unique identifiers, or web links for publicly available datasets
- A description of any restrictions on data availability
- For clinical datasets or third party data, please ensure that the statement adheres to our policy

Provide your data availability statement here.

Field-specific reporting

Please select the one below that is the best fit for your research. If you are not sure, read the appropriate sections before making your selection.

☒ Life sciences ☐ Behavioural & social sciences ☐ Ecological, evolutionary & environmental sciences

For a reference copy of the document with all sections, see nature.com/documents/nr-reporting-summary-flat.pdf

Life sciences study design

All studies must disclose on these points even when the disclosure is negative.

Sample size	Sample sizes were chosen using G*Power 3.1 with alpha=0.5, power (1-beta)=0.8 to detect at least a 70% effect compared to control group
Data exclusions	No data were excluded.
Replication	All animals used appropriate group sizes to determine
Randomization	All animals were randomly assigned into groups by the commercial vendors prior to arrival.
Blinding	For scoring of animals in challenge studies, researchers were blinded to the treatments. For histopathological analysis, pathologists were blind to the treatments.

Reporting for specific materials, systems and methods

We require information from authors about some types of materials, experimental systems and methods used in many studies. Here, indicate whether each material, system or method listed is relevant to your study. If you are not sure if a list item applies to your research, read the appropriate section before selecting a response.

Materials & experimental systems

n/a	Involved in the study	
☐	☒	Antibodies
☐	☒	Eukaryotic cell lines
☒	☐	Palaeontology and archaeology
☐	☒	Animals and other organisms
☒	☐	Human research participants
☒	☐	Clinical data
☒	☐	Dual use research of concern

Methods

n/a	Involved in the study	
☒	☐	ChIP-seq
☒	☐	Flow cytometry
☒	☐	MRI-based neuroimaging

Antibodies

Antibodies used	Some antibodies were made in-house, and relevant references to sequences are included in the manuscript. Secondary antibodies used are as follows: Goat anti-Mouse IgG (H+L) Cross-Adsorbed Secondary Antibody, HRP (Invitrogen Catalog # A16072). Goat anti-Human IgG (H+L) Cross-Adsorbed Secondary Antibody, HRP (Invitrogen Catalog # 31412).
Validation	In-house made antibodies were validated by ELISA for binding to known epitopes and were validated in depth in the original research articles describing them. Goat anti-human IgG supplier's website states "This antibody has been successfully used in Western blot, and ICC applications.<br><br>Antibody Specificity: When tested by immunoelectrophoresis, this antibody reacts with the heavy chains of human IgG and with light chains common to most human immunoglobulins. No antibody was detected against non-immunoglobulin serum proteins. The antibody has been tested by ELISA and/or solid-phase adsorbed to ensure minimal cross-reaction with bovine, horse and mouse serum proteins. However, this antibody may cross-react with immunoglobulins from other species." The supplier of the goat anti-mouse HRP secondary antibody states the antibody was validated via Western blotting using an anti-SOD1 mouse monoclonal antibody.

Eukaryotic cell lines

Policy information about cell lines

Cell line source(s)	Cells were purchased from the ATCC or commercial suppliers as indicated in the manuscript.
Authentication	None of the cell lines used were authenticated

Mycoplasma contamination	Cell lines were regularly tested for mycoplasma contamination and were negative.
Commonly misidentified lines (See ICLAC register)	*Name any commonly misidentified cell lines used in the study and provide a rationale for their use.*

Animals and other organisms

Policy information about studies involving animals; ARRIVE guidelines recommended for reporting animal research

Laboratory animals	This study utilised female C57BL/6 mice and male and female ferrets.
Wild animals	This study did not involve wild animals
Field-collected samples	This study did not collect samples from the field.
Ethics oversight	All animal experiments were approved by the relevant animal ethics committees at either the University of Queensland (AEC number SBMS/071/17 or the University of Melbourne (AEC number 1714278.4). The ferret experiments carried out at Viroclinics Xplore in Schaijk, The Netherlands, were under conditions that meet the standard of Dutch law for animal experimentation. The facility is fully accredited by the Dutch ministry that governs and inspects the animal facilities and oversees, coordinates and inspects activities of the animal ethics committees of Dutch institutions and academic centres. A registered article 9 officer is responsible for the design and management of the experiments, in close consultation with the animal welfare body (IvD) who grated ethical approval for the experiments, registered under Working Protocol number: AVD277002015142-1-WP21.

Note that full information on the approval of the study protocol must also be provided in the manuscript.
